# Interactional Effects of Climate Change Factors on the Water Status, Photosynthetic Rate, and Metabolic Regulation in Peach

**DOI:** 10.3389/fpls.2020.00043

**Published:** 2020-02-28

**Authors:** Sergio Jiménez, Masoud Fattahi, Khaoula Bedis, Shirin Nasrolahpour-moghadam, Juan José Irigoyen, Yolanda Gogorcena

**Affiliations:** ^1^ Laboratory of Genomics, Genetics and Breeding of Fruit Trees and Grapevine, Department of Pomology, Estación Experimental de Aula Dei-Consejo Superior de Investigaciones Científicas, Zaragoza, Spain; ^2^ Bayer AG, Crop Science Division, Research and Development, Environmental Science Field Solutions, Monheim, Germany; ^3^ Department of Agriculture, Shahrekord University, Shahrekord, Iran; ^4^ Departamento de Biología Ambiental, Grupo de Fisiología del Estrés en Plantas, Unidad Asociada al CSIC (EEAD, Zaragoza e ICVV, Logroño), Facultad de Ciencias, Universidad de Navarra, Pamplona, Spain

**Keywords:** *Prunus* rootstocks, elevated CO_2_, warming, drought, osmotic potential, water-use efficiency, soluble sugars, gene expression

## Abstract

Environmental stress factors caused by climate change affect plant growth and crop production, and pose a growing threat to sustainable agriculture, especially for tree crops. In this context, we sought to investigate the responses to climate change of two *Prunus* rootstocks (GF677 and Adesoto) budded with Catherina peach cultivar. Plants were grown in 15 L pots in temperature gradient greenhouses for an 18 days acclimation period after which six treatments were applied: [CO_2_ levels (400 versus 700 µmol mol^-1^), temperature (ambient versus ambient + 4°C), and water availability (well irrigated versus drought)]. After 23 days, the effects of stress were evaluated as changes in physiological and biochemical traits, including expression of relevant genes. Stem water potential decreased under drought stress in plants grafted on GF677 and Adesoto rootstocks; however, elevated CO_2_ and temperature affected plant water content differently in both combinations. The photosynthetic rate of plants grafted on GF677 increased under high CO_2_, but decreased under high temperature and drought conditions. The photosynthetic rates of plants grafted onto Adesoto were only affected by drought treatment. Furthermore, in GF677–Catherina plants, elevated CO_2_ alleviated the effect of drought, whereas in those grafted onto Adesoto, the same condition produced acclimation in the rate. Stomatal conductance decreased under high CO_2_ and drought stress in both grafted rootstocks, and the combination of these conditions improved water-use efficiency. Changes in the sugar content in scion leaves and roots were significantly different under the stress conditions in both combinations. Meanwhile, the expression of most of the assessed genes was significantly affected by treatment. Regarding genotypes, GF677 rootstock showed more changes at the molecular and transcriptomic level than did Adesoto rootstock. A coordinated shift was found between the physiological status and the transcriptomic responses. This study revealed adaptive responses to climate change at the physiological, metabolic, and transcriptomic levels in two *Prunus* rootstocks budded with 'Catherina'. Overall, these results demonstrate the resilient capacity and plasticity of these contrasting genotypes, which can be further used to combat ongoing climate changes and support sustainable peach production.

## Introduction

Peach is the third most important temperate fruit tree species of the Rosaceae family, behind apples and pears (FAOSTAT, 2018; http://faostat.fao.org), with China being the largest producer (14.3 million tons), followed by European countries (Spain, Italy, and Greece) and the United States. In 2017, world growth area and production were 1.52 million hectares and 24.7 million tons, respectively. Peach is grown in temperate areas and it is routinely grafted on rootstocks for adaption to different soil and climate conditions. Predictions for new climate scenarios, which include an increase in temperature, alterations in rainfall patterns, and increasing frequency of extreme climate events, are likely to negatively affect global agriculture, especially in Mediterranean regions (IPCC, 2014; FAO, 2016; IPCC, 2018). This concern is especially relevant for peach trees because warming temperatures will impact negatively flowering and production (Gogorcena et al., 2020). In this situation, it is still more critical to choose the correct rootstock–scion combination to cope with the effects of climate change.

In the last century, climate change (high CO_2_ concentration and temperature, and limited availability of water) has become a major concern for the scientists. According to long-term warming trends since pre-industrial times, temperatures are estimated to have increased by 0.1 to 0.3°C per decade across the world (IPCC, 2014; IPCC, 2018) and the mean global temperature is expected to increase by 1.5°C between 2030 and 2052 if it continues to rise at the current rate (IPCC, 2018). Atmospheric CO_2_ concentrations have risen at an accelerated pace since the start of the industrial revolution. For the one thousand years prior to the industrial revolution, CO_2_ levels were stable at about 280 µmol mol^-1^. Nowadays, this concentration is approximately 53% higher at 414 µmol mol^-1^ (NOAA Mauna Loa Atmospheric Baseline Observatory, 2019). By the end of this century, it is predicted to reach 700 µmol mol^-1^ (Long et al., 2004; Ainsworth et al., 2008; Salazar-Parra et al., 2018). To cope with such catastrophic climate change, plants need to develop a width spectrum of physiological, biochemical, and molecular programs to rapidly sense change and adapt. In this context, understanding how peach may respond and adapt to future increases in CO_2_ concentration, temperature, and drought is critical for the agricultural fruit sector.

Previous studies have shown that elevated CO_2_ concentrations stimulate photosynthetic carbon gain and net primary production (Leakey et al., 2009; Medina et al., 2016; Afzal et al., 2018). However, in long-term experiments, it has been reported that the initial stimulation of photosynthesis decreases due to acclimation of photosynthetic capacity (Leakey et al., 2009; Aranjuelo et al., 2011; Salazar-Parra et al., 2015; Medina et al., 2016), and that environmental or genetic factors predispose plants to greater or lesser variation (reviewed in Arp, 1991; Leakey et al., 2009 and references therein; Medina et al., 2016). Moreover, elevated CO_2_ improves nitrogen-use efficiency and decreases water use in leaves (Medina et al., 2016). Furthermore, elevated CO_2_ stimulates leaf dark respiration *via* a transcriptional reprogramming of metabolism in soybean, but not in other species (Leakey et al., 2009).

It is well accepted that water scarcity will dramatically increase due to climate change and will become a major problem for crop production by limiting the growth and productivity of many crop species. Water limitation in the near future has resulted in strong interest in drought tolerance afforded by rootstocks (Serra et al., 2014), which enable the scion to grow and bear fruit. Plant responses to water limitation are usually monitored through select morphological and physiological traits (Jiménez et al., 2013; Santana-Veira et al., 2016; Fathi et al., 2017; Afzal et al., 2018). Drought inhibits the growth and development of plants, directly affecting the photosynthetic process, resulting in physiological limitations and transcriptional responses that may cause severe decreases in plant yield (Jiménez et al., 2013; Nakashima et al., 2014; Ksouri et al., 2016). Under these stress conditions, there are physiological changes such as reduction of net photosynthesis, and decreases in stomatal conductance and internal CO_2_ concentrations (Jones, 2007; Cattivelli et al., 2008; Lovisolo et al., 2010). The decrease of stomatal conductance may lead to the reduction of transpiration and water losses as well as to overproduction of reactive oxygen species (ROS) and activation of antioxidant enzymes (Gogorcena et al., 1995; Salazar-Parra et al., 2012; Haider et al., 2017). The accumulation of metabolites such as soluble carbohydrates and proline in leaves and roots of *Prunus* (Jiménez et al., 2013; Haider et al., 2018), carbohydrates and proline in leaves of pearl millet (Fabbrin et al., 2015), and carbohydrates in citrus rootstocks (Pedroso et al., 2014; Santana-Veira et al., 2016) have all been reported previously as a consequence of drought stress.

Plant responses to elevated CO_2_ in combination with drought stress and/or temperature increases have been widely studied in different plant species, such as wheat (Erice et al., 2014; Medina et al., 2016), grapevines (Kizildeniz et al., 2015; Salazar-Parra et al., 2015; Martínez-Lüscher et al., 2016; Kizildeniz et al., 2018), alfalfa, soybean, and other plant species (Aranjuelo et al., 2005; Aranjuelo et al., 2006; Baslam et al., 2014; Gray and Brady, 2016 and references therein; Irigoyen et al., 2014; Kelly et al., 2016). These earlier studies have found that responses are genotype-dependent; however, conflicting experimental results make it difficult to draw general conclusions (Kelly et al., 2016). Some studies have shown positive effects of elevated CO_2_ on water stress tolerance in some wheat and grapevine genotypes, but these effects were not universal. In wheat, elevated CO_2_ promoted plant growth and mitigated the deleterious effect of drought on biomass decreases (Medina et al., 2016). In grapevines, a protective effect of CO_2_ independent of temperature was found concerning oxidative damage (Salazar-Parra et al., 2015) and plant growth (Kizildeniz et al., 2015). However, in bread wheat genotypes, the effect of CO_2_ and drought interacted to cause oxidative stress (Bencze et al., 2014), and in other woody species, such as American sycamore, sweet gum, and sugar maple, it negatively affected growth (Gray and Brady, 2016). Other changes under elevated CO_2_ and drought stress have been described, such as a decrease in Rubisco content and activity, changes in amino acids and N content, and an increase in carbohydrates in wheat (Aranjuelo et al., 2011), as well as increases in sugar and changes in organic acids and anthocyanin content in grape berries (Kizildeniz et al., 2015).

In fruit plant breeding, rootstocks have been shown to play an important role in drought tolerance by adjusting the water supply to the demands of shoot transpiration. In fact, rootstocks are considered to confer drought and heat tolerance to the scion (Iacono et al., 1998; Meggio et al., 2014; Serra et al., 2014). Morphological and physiological changes were observed in *Prunus* rootstocks subjected to water deprivation (Solari et al., 2006; Jiménez et al., 2013). Apart from these changes, water-stressed plants may accumulate proline and raffinose in leaves and roots to protect membranes and enzymes, and to deal with the deleterious effects of drought-induced oxidative stress (Jiménez et al., 2013). Proline content in roots and leaves, sorbitol in leaves, and raffinose in roots were all found to be associated with increases in water-use efficiency (Jiménez et al., 2013). Moreover, at the transcriptional level, changes in gene expression were consistently found to support the accumulation of these metabolites in root and leaf tissues in *Prunus* (Jiménez et al., 2013) and in grapevines (Haider et al., 2017).

As mentioned above, agriculture productivity is strongly affected by drought, temperature increases, and other forms of climate changes (FAO, 2016; Afzal et al., 2018). In the future, plants will not experience individual climate change factors, but will be exposed to several interacting environmental effects at the same time (Gray and Brady, 2016). Plant responses to elevated CO_2_, temperature, and drought are genotype-dependent (Kizildeniz et al., 2015; Medina et al., 2016; Kizildeniz et al., 2018) and the interactive effects of environmental conditions and genotypic influences cannot be anticipated by studying the effect of each individual climate change factor. For this reason, to investigate plant responses where CO_2_ concentration, temperature, and water availability can be modulated simultaneously, gradient temperature greenhouses are needed to enable comparisons of current climate with future predictions (Morales et al., 2014). Previous investigations in these facilities have been carried out by a number of authors for different herbaceous plant species (Sanz-Sáez et al., 2012; Sanz-Sáez et al., 2013; Fabbrin et al., 2015) and grapevines (Kizildeniz et al., 2015; Salazar-Parra et al., 2015; Martínez-Lüscher et al., 2016; Kizildeniz et al., 2018; Salazar-Parra et al., 2018), but never for *Prunus* spp and never taking into consideration rootstock plasticity.

The aim of the present work is to investigate the physiological, biochemical, and molecular responses of two contrasting *Prunus* rootstocks (GF677 and Adesoto) budded with Catherina peach cultivar to climate change-induced stresses (elevated CO_2_, elevated temperature, and water deficit). Understanding how rootstocks with different genetic background modulate the response of peach trees under stress conditions and disentangle the underlying molecular mechanisms will be very helpful to develop resilient rootstocks in future breeding programs.

## Materials and Methods

### Plant Material and Experimental Conditions

Micropropagated GF677 (*Prunus dulcis* Miller × *P. persica* L. Batsch) and Adesoto (*P. insititia* L.) rootstock plants were grown for two weeks in 300 cm^3^ pots containing a peat substrate, then they were micrografted with variety Catherina (*P. persica* L. Batsch). Plants were transferred to 15 L containers with a medium of 1:1 sand-peat substrate (TKS-1, Floragard, Oldenburg, Germany) and 2 g kg^−1^ osmocote 14-13-13 (The Scotts Company LLC, Marsyville, OH, USA). Plants were grown for two months in an experimental greenhouse in Zaragoza, Spain (41°43′N, 0°48′W) under normal day light conditions (14 h light/10 h dark photoperiod) with mean day and night temperatures and humidity of 24 and 18°C, and 51 and 67%, respectively. Plants were divided randomly into eight groups (20 plants per group) and were transferred to four greenhouses and grown at the University of Navarra (Pamplona, Spain, 42°48′N, 1°40′W). All temperature gradient greenhouses (TGGs) have been designed in a modular way to have a temperature gradient (ambient to + 4°C) and CO_2_ gassed inside to reach the desired CO_2_ concentration. Treatments were a combination of two CO_2_ levels (ambient, approximately 400 ppm and elevated, 700 ppm), two temperature regimes (ambient temperature and ambient + 4°C) for 18 d, a period that used for acclimation. Then plants were subjected to two regimes of irrigation. Well-watered plants were maintained at around 80% of the substrate field capacity. In the water-deficit treatment, plants were watered daily with the 80% of the evapotranspirated water (Jiménez et al., 2013). Then, in each greenhouse, plants were irrigated and partially irrigated for 23 d. Soil water sensors (Watermark soil moisture sensor, Spectrum Technologies Inc., IL, USA) were placed into the pots and used for irrigation control. Stems (leaves and main shoot) and roots were harvested at 23 d, weighed (fresh weight, FW), and then oven-dried (dry weight, DW) at 80°C for 48 h. For all treatments, specific leaf area (SLA) was measured and chlorophyll (Chl) concentration per unit leaf area was estimated using a SPAD 502 meter (Minolta Co., Osaka, Japan). Samples from plants submitted to ambient and elevated CO_2_, ambient and elevated temperature, and control and drought stress for 23 d were randomly collected. Root and leaf tissues from each treatment-plant (four biological replicates) were rinsed in distilled water, immediately frozen in liquid nitrogen and stored at -80°C until their use for the molecular determinations.

### Water Status

A single mature leaf (fifth expanded leaf) of each of the four replicate plants was assayed for stem water potential (Ψstem) at day 23 of the experiment. Leaves were enclosed in aluminum foil-covered plastic envelopes to stop transpiration and allow equilibration with Ψstem 30 min before measurement. Midday Ψstem were measured using a Schölander-type pressure chamber (PMS instrument, Corvallis, OR, USA). After measurements, leaves were wrapped in aluminum foil, frozen in liquid nitrogen, and stored in plastic bags at -20°C (García-Sánchez et al., 2007). After thawing, osmotic potential (Ψπ) was measured with a Psychrometer Tru PSi SC10X (Decagon Devices, Inc., Pullman, WA, USA).

Leaf relative water content (RWC) was measured on a mature leaf (sixth expanded leaf) of the four replicate plants. Leaves were immediately weighed to obtain a leaf FW and petioles were submerged into water overnight in the dark. Fully hydrated leaves were reweighed to obtain turgid weight (TW) and dried at 80°C for 24 h to obtain DW. RWC was calculated as 100×(FW-DW)/(TW-DW) according to Morgan (Morgan, 1984).

### Photosynthetic Parameters

Photosynthetic rate (*A*_N_), stomatal conductance (*g*_s_), intercellular CO_2_ concentration (*C*i), and transpiration rate (*E*) were measured after 23 d using a portable photosynthesis system (LI-6400XT, Licor, Inc., Lincoln, NE, USA). Measurements were conducted between 10:00 and 12:00 (GMT) in the same leaves used for Ψstem determinations (n = 4). Parameters were measured with saturating light (1400 μmol m^−2^ s^−1^ provided by an external light source), 400 μmol mol^−1^ CO_2_ and 30.5°C (average leaf temperature during measurements). Water-use efficiency (WUE) or instantaneous water-use efficiency was calculated as the ratio between the photosynthetic rate and stomatal conductance (*A*_N/_ *g*_s_).

### Osmotic-Regulating Compounds: Soluble Sugars and Proline

Leaf and root soluble sugar content was determined by high-performance liquid chromatography (HPLC). Plant tissue (n = 4) was ground to a fine powder in a pre-cooled mortar with liquid nitrogen. Polar compounds from ~0.1 g FW were extracted into aqueous ethanol at 80°C, in three steps, each lasting 20 min (step 1: 0.75 ml of 80% ethanol; steps 2 and 3: 0.75 ml of 50% ethanol). The mixture of each step was centrifuged for 10 min at 4800*g* and slurries were pooled (Moing et al., 2004). The ethanol was allowed to evaporate in a speed-vac and dry extracts were solubilized in 1 ml double-distilled water. Soluble sugars were purified using ion exchange resins (Bio-Rad AG 1-X4 Resin 200-400 chloride form, Bio-Rad AG 50W-X8 Resin 200-400 mesh hydrogen form, Bio-Rad, Hercules, CA, USA). Samples were concentrated to 0.2 ml, filtered and 20 μl was injected and analyzed by HPLC, using a Ca-column (Aminex HPX-87C 300 mm × 7.8 mm column Bio-Rad) flushed with 0.6 ml min^−1^ double-distilled water at 85°C with a refractive index detector (Waters 2410) (Milford, MA, USA). Concentrations of the main sugars (fructose, glucose, raffinose, sorbitol, sucrose, and xylose) were calculated for each sample, using mannitol as an internal standard. Sugar quantification was carried out with Empower Login software from Waters, using commercial standards (Panreac Química S.A. Barcelona, Spain). The amounts of soluble sugars were reported as mg g^−1^ DW.

Leaf and root proline were determined using the methodology described previously (Bates et al., 1973; Ábrahám et al., 2010). Plant tissue (n = 4) was ground to a fine powder in a pre-cooled mortar with liquid nitrogen. About 0.1 g of FW per sample was homogenized with 3% sulfosalicylic acid (Panreac Química S.A.) and the supernatant was reacted with ninhydrine (Sigma-Aldrich, St. Louis, MO, USA). The absorbance was read at 520 nm and the free proline concentration was calculated from a calibration curve using proline as a standard (Sigma-Aldrich). Free proline content was reported as mg g^−1^ DW.

### RNA Isolation and RT-qPCR

Frozen plant tissue (four biological replicates) was ground to a fine powder in a pre-cooled mortar with liquid nitrogen and subsequently total RNA was isolated from ~100 mg of FW following the protocol of Meisel et al. (2005) with some modifications. After DNase I treatment (Thermo Scientific, Waltham, MA, USA) to eliminate possible genomic DNA contamination, 2 μg of total RNA were reverse transcribed using an oligo-(dT) 18 as a primer with RevertAid H Minus first-strand cDNA synthesis system (Thermo Scientific). Samples from cDNA synthesis were used to evaluate the expression of genes involved in sorbitol metabolism and raffinose and proline synthesis. These included sorbitol dehydrogenase (*SDH*), sorbitol-6-phosphate dehydrogenase (*S6PDH*), raffinose synthase (*SIP1*), Δ-1-pyrrolyne-carboxylate synthase (*P5CS*), Δ-1-pyrrolyne-carboxylate reductase (*P5CR*), and ornithine aminotransferase (*OAT*), which encodes an enzyme that synthesizes a precursor for proline biosynthesis. Also, phosphatidylinositol 4,5-bisphosphate (*PIP2*), which plays a role in membrane transport, dehydration responsive element binding protein (*DREB2*), ABA responsive element binding protein (*AREB2*), and the homeodomain-leucine zipper protein (*HAT22*) genes were assayed (**Supplementary Table 1**). Gene sequences were identified by Blastn against the “Peach Genome v1.0 predicted transcripts” database in GDR (http://www.rosaceae.org) with an *E*-value of >1 × 10^−5^. Finally, gene-specific primers were designed using Primer3Plus (Untergasser et al., 2007). Real-time qPCR was carried out using the Kapa SYBR Fast Maxter Mix (Kapa Biosystems, Cambridge, MA, USA) on a Applied Biosystem 7500 Real Time PCR (Life Technologies, Carlsbad, CA, USA) as described previously (Ksouri et al., 2016). Fluorescence values were baseline-corrected and averaged efficiencies for each gene and quantification cycle (Cq) values were calculated using LinRegPCR program (Ruijter et al., 2009). Gene expression was determined with the gene expression Cq difference (GED) formula (Schefe et al., 2006) using *Actin 2* as an internal reference gene. Gene expression levels were normalized relative to the values of the drought-tolerant GF677 under control conditions (Jiménez et al., 2013; Ksouri et al., 2016). Normalized data allowed for the comparison of the magnitude of gene expression both across treatments and genotypes.

### Statistical Analysis

Data were evaluated by three-way (2 CO_2_ × 2 temperature × 2 water regimes) analysis of variance (ANOVA) for each genotype-tissue with SPSS 25.0.0 (Inc., Chicago, IL, USA). Previously, data were normalized and evaluated by Levene's homoscedasticity test and transformed if necessary. The main treatment parameters (CO_2_, temperature, and drought) were evaluated alone and as interactions. For simplicity in figures, only two-level interactions (CO_2_ × T^e^) or triple (CO_2_ × T^e^ × drought) were labeled. When treatment interaction terms were significant (*P *≤ 0.05), means were separated using Duncan's multiple range test at *P ≤* 0.05. Means of two samples were compared using a Student t-test. Regression analysis was carried out by Pearson's correlation.

## Results

### Effect of Climate Change on Biomass, Water Status and Physiological Traits

#### Biomass

After 23 d of treatment, elevated CO_2_ and drought modified biomass in plants grafted on both genotypes whereas temperature did not affect plant growth. High CO_2_ concentrations increased leaf and root DW only in grafted GF677 plants, but decreased shoot/root DW ratio in both genotypes (**Table 1**). In GF677–Catherina plants, elevated CO_2_ decreased specific leaf area, while drought decreased the shoot/root ratio and increased SPAD values. Drought decreased leaf DW in both genotypes and, as a consequence, the shoot/root ratio also decreased.

**Table 1:** Leaf and root dry weight (DW), shoot-to-root ratio, specific leaf area (SLA), and SPAD in control and stressed *Prunus* rootstocks (GF677 and Adesoto) budded with var. Catherina, after 23 days of treatment.

GF677		Leaf DW (g)		Root DW (g)		Shoot/Root DW ratio		SLA(cm^2^ g^-1^ DW)		SPAD	
**Principal Effects**											
CO_2_	CO_2_ Amb	4.8	b	3.0	b	3.3	a	166	a	44	
	CO_2_ Elev	6.3	a	4.9	a	2.7	b	145	b	44	
											
Temperature	T^e^ Amb	5.0		3.7		2.9		150		45	
	T^e^ Amb+4°C	6.0		4.3		3.2		160		44	
											
Irrigation	Control	6.6	a	4.3		3.4		159		43	b
	Drought	4.4	b	3.6		2.7		151		46	a
***Signification***											
CO_2_		*		**		*		***		ns	
T^e^		ns		ns		ns		ns		ns	
Irrigation		**		ns		*		ns		***	
**Adesoto**											
**Principal Effects**											
CO_2_	CO_2_ Amb	4.1		2.9		3.0	a	157		43	
	CO_2_ Elev	3.6		3.5		2.4	b	147		42	
											
Temperature	T^e^ Amb	3.6		3.1		2.5		146		42	
	T^e^ Amb+4°C	4.1		3.2		3.0		158		42	
											
Irrigation	Control	4.7	a	3.2		3.0	a	155		42	
	Drought	3.0	b	3.2		2.4	b	149		42	
***Signification***											
CO_2_		ns		ns		*		ns		ns	
T^e^		ns		ns		ns		ns		ns	
Irrigation		***		ns		*		ns		ns	

Three-way ANOVA was performed for linear model, on raw data. Significance: *P ≤ 0.05, **P ≤ 0.01, ***P ≤ 0.001 and ns indicates not significant. Comparison means by Duncan's test (P ≤ 0.05) were shown for the significant interaction among treatments. Different letters indicate significant differences among data within the same factor. Amb, Ambient; Elev, Elevated; T^e^, Temperature. These data were presented at the conference of the Spanish Society of Plant Physiology (Fattahi et al., 2019).

#### Stem Water and Osmotic Potentials

**Figure 1:**
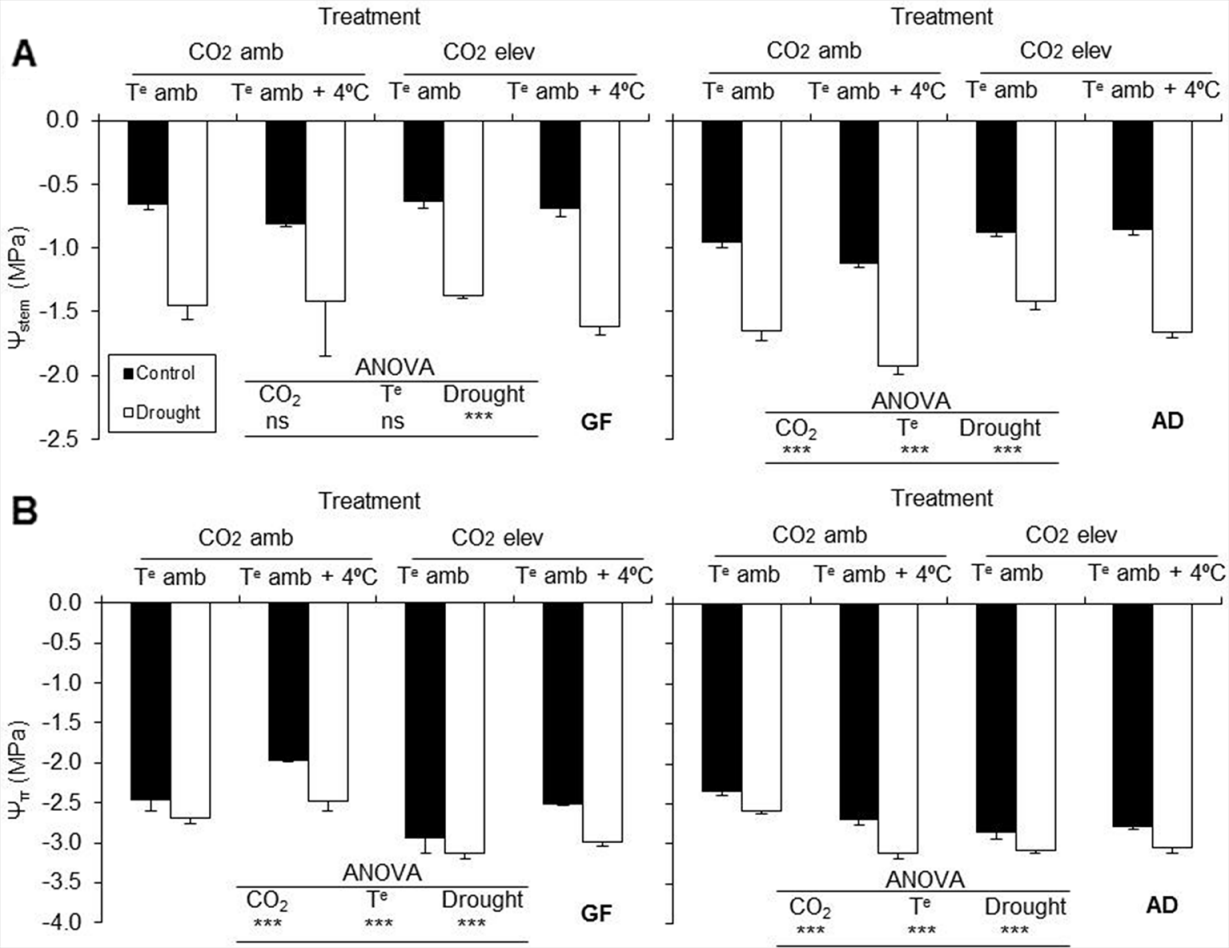
**(A)** Stem water potential (Ψstem) and **(B)** osmotic potential (Ψπ) in peach plants (variety Catherina) grafted on GF677 (GF) and Adesoto (AD) and subjected to ambient (amb CO_2_) and high (CO_2_ elev) CO_2_, ambient (T^e^ amb) and high (T^e^ amb + 4°C) temperature, and control irrigation and drought for 23 days. Vertical bars indicate the standard error (n = 4). Significant differences: *** *P* ≤ 0.001 and ns: non-significant.

Drought stress reduced stem water and osmotic potentials in Catherina cv. grafted on both rootstocks, GF677 and Adesoto (*P ≤* 0.001). The Ψstem in Catherina cv. grafted on Adesoto was also affected by the CO_2_ concentration and temperature (*P ≤* 0.001) (**Figure 1A**). The osmotic potential, Ψπ, was significantly diminished by elevated CO_2_ and affected by temperature (*P ≤* 0.001) in plants grafted on both rootstocks (**Figure 1B**). Elevated CO_2_ in plants grafted on Adesoto and elevated temperature in those grafted on GF677 increased stem water and osmotic potentials, respectively.

#### Photosynthetic Response and Gas Exchange

**Figure 2:**
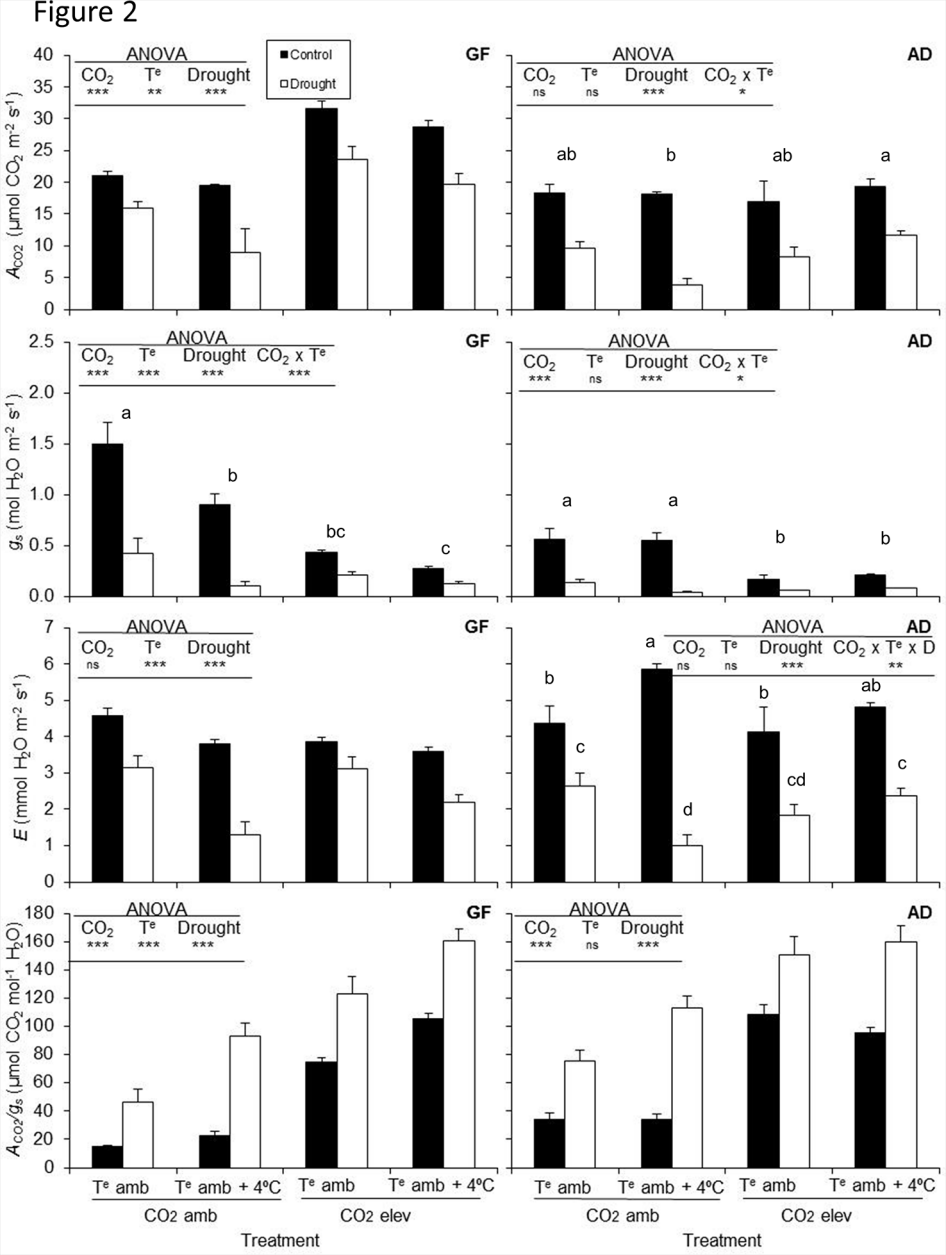
Photosynthetic rate (*A_N_*), stomatal conductance (*g*_s_), transpiration rate (*E*), and water-use efficiency (*A*_N_ */g*_s_) in peach plants (variety Catherina) grafted on GF677 (GF) and Adesoto (AD) and subjected to ambient (CO_2_ amb) and high (CO_2_ elev) CO_2_, ambient (T^e^ amb) and high (T^e^ amb + 4°C) temperature, and irrigation control (C), and drought (D) for 23 days. Vertical bars indicate the standard error (n = 4). Significant differences: * *P* ≤0.05, ** *P* ≤0.01, *** *P* ≤0.001 and ns: non-significant. For the significant double (CO_2_ × T^e^) and triple (CO_2_ × T^e^ × D) interactions, differences among means are shown with different letters (Duncan's test, *P < *0.05*)*.

The photosynthetic rate (*A*_N_) of plants grafted on GF677 increased under elevated CO_2_, but decreased with high temperature and drought (**Figure 2**). However, when plants were grafted onto Adesoto rootstock, the photosynthetic rate decreased only under drought stress condition. Stomatal conductance (*g*_s_) of grafted plants on GF677 and Adesoto rootstocks decreased with elevated CO_2_ concentration and drought stress (**Figure 2**), while transpiration rate (*E*) decreased only under drought stress condition. Elevated temperature did not affect the Adesoto rootstock.

In both genotypes, the climate change-like conditions, except for elevated temperature in Adesoto, improved the WUE (**Figure 2**).

Interactive effects among treatments were found for both grafted GF677 and Adesoto rootstocks, between CO_2_ and temperature (*A*_N_ in Adesoto, and *g*_s_ in both genoypes, **Figure 2**), between CO_2_ and drought (*A*_N_, *E* in GF677, and *g*_s_ in both genotypes), and between temperature and drought (*E* and WUE in both genotypes). A triple interaction was found only in Adesoto–Catherina for transpiration rate (*E*, **Figure 2**). Interaction between elevated temperature and drought resulted in a higher WUE in drought stressed plants in both genotypes.

### Effect of Climate Change on Soluble Carbohydrates and Proline Content

The biochemical responses involving sugars and proline content in roots and leaves of 'Catherina' grafted on both rootstocks (GF677 and Adesoto) subjected to stresses associated with climate change for 23 d are shown in **Tables 2** and **3**. In roots, elevated CO_2_ increased the concentration of glucose and total sugars in both rootstocks, xylose in GF677, and fructose in Adesoto (**Table 2**). In this study, we noticed that the content of sorbitol and total sugars increased in response to high temperature, but only in roots of Adesoto (**Table 2B**). In roots, under drought stress condition, raffinose and proline concentration increased in GF677, sorbitol decreased in GF677, but increased in Adesoto rootstock, and fructose decreased in Adesoto (**Table 2**). The interactive effects between treatments also significantly increased the concentration of sugars (**Supplementary Tables 2** and **3**). In GF677 rootstock, the interaction between elevated temperature and irrigation increased root raffinose and xylose concentration. In contrast, in Adesoto rootstock, the interaction between elevated CO_2_ and irrigation increased root glucose content.

**Table 2:** Root soluble sugars and proline (mg g^-1^ DW) concentration (n=4) in ambient (amb CO_2_) and high (CO_2_ elev) CO_2_, ambient (T^e^ amb) and high (T^e^ amb + 4°C) temperature, and control irrigation and drought-stressed GF677 (A) and Adesoto (B) *Prunus* rootstocks budded with cv. Catherina, after 23 days of treatment.

Roots		Fructose	Glucose	Raffinose	Sucrose	Sorbitol	Xylose	Total sugars	Proline
A) GF677									
**Principal Effects**									
CO_2_	CO_2_ Amb	4.5	11.9 b	1,3	17.6	14.2	0.6 b	50.1 b	1.0
	CO_2_ Elev	5.4	20.0 a	1,2	21.0	13.7	1.0 a	62.2 a	1.3
T^e^	T^e^ Amb	4.4	14.6	1,2	20.5	13.6	0.9	55.1	1.0
	T^e^ Amb+4°C	5.6	17.5	1,3	18.2	14.2	0.8	57.5	1.4
Irrigation	Control	4.7	14.6	0.9 b	18.3	16.2 a	0.8	55.5	0.7 b
	Drought	5.3	17.4	1.6 a	20.3	11.7 b	0.8	57.1	1.7 a
***Signification***									
CO_2_		ns	***	ns	ns	ns	**	**	ns
T^e^		ns	ns	ns	ns	ns	ns	ns	ns
Irrigation		ns	ns	**	ns	***	ns	ns	***
CO_2_ × T^e^		ns	ns	ns	ns	ns	ns	ns	ns
CO_2_ × Irrigation		ns	ns	ns	ns	ns	ns	ns	ns
T^e^ × Irrigation		ns	ns	**	ns	ns	*	ns	ns
CO_2_ × T^e^ × Irrigation		ns	ns	ns	ns	ns	ns	ns	ns
				**B) Adesoto**						
**Principal Effects**									
CO_2_	CO_2_ Amb	4.7 b	18.7 b	0.6	26.3	18.6	0.9	70.5 b	0.9
	CO_2_ Elev	5.8 a	23.6 a	0.7	30.5	20.3	1.1	82.7 a	1.0
T^e^	T^e^ Amb	5.0	21.4	0.7	27.4	15.3 b	0.9	71.3 b	0.9
	T^e^ Amb+4°C	5.4	21.0	0.7	29.5	23.3 a	1.1	81.4 a	1.0
Irrigation	Control	5.9 a	19.7	0.6	30.7	14.2 b	1.1	72.6	0.9
	Drought	4.6 b	22.6	0.8	26.4	24.4 a	1.0	80.2	1.0
***Signification***									
CO_2_		**	*	ns	ns	ns	ns	*	ns
T^e^		ns	ns	ns	ns	***	ns	*	ns
Irrigation		**	ns	ns	ns	***	ns	ns	ns
CO_2_ × T^e^		ns	ns	ns	ns	ns	ns	ns	ns
CO_2_ × Irrigation		ns	**	ns	ns	ns	ns	ns	ns
T^e^ × Irrigation		ns	ns	ns	ns	ns	ns	ns	ns
CO_2_ × T^e^ × Irrigation		ns	ns	ns	ns	ns	ns	ns	ns

Three-way ANOVA was performed for linear model, on raw data. Significance: *P ≤ 0.05, **P ≤ 0.01, ***P ≤ 0.001 and ns indicates not significant. Comparison means by Duncan's test (P ≤ 0.05) were shown for the significant interaction among treatments. Different letters indicate significant differences among data within the same factor. Amb, Ambient; Elev, Elevated; T^e^, Temperature.

Concerning leaves of 'Catherina' grafted on GF677, elevated CO_2_ significantly increased the concentration of all sugars except xylose, while leaves of ‘Catherina’ grafted on Adesoto rootstock showed increases only for sucrose (**Table 3**). Elevated temperature affected only leaves of the GF677–Catherina combination, increasing the content of glucose and decreasing the content of sucrose and proline. Drought had the same effects for sucrose, xylose, and proline in leaves of both combinations. Under drought stress, sucrose decreased, while xylose and proline increased. Furthermore, in the GF677–Catherina combination, this stress condition led to decreased fructose and increased sorbitol content. Interactive effects of CO_2_ with irrigation in GF677–Catherina led to increases in leaf sorbitol and proline, but in Adesoto–Catherina, the triple interaction (CO_2_ × temperature × drought) seemed to maintain the levels of sucrose, sorbitol, and total sugars (**Supplementary Tables 4** and **5**).

**Table 3:** Scion leaf soluble sugars and proline (mg g^-1^ DW) concentration (n = 4) in ambient (amb CO_2_) and high (CO_2_ elev) CO_2_, ambient (T^e^ amb) and high (T^e^ amb + 4°C) temperature, and control irrigation and drought-stressed GF677 (A) and Adesoto (B) *Prunus* rootstocks budded with cv. Catherina, after 23 days of treatment.

Leaves		Fructose	Glucose	Raffinose	Sucrose	Sorbitol	Xylose	Total sugars	Proline
				A) GF677					
**Principal Effects**									
CO_2_	CO_2_ Amb	8.4 b	11.1 b	0.3 b	40.2 b	80.6 b	1.2	142.1 b	1.7 b
	CO_2_ Elev	10.3 a	14.2 a	0.4 a	60.5 a	90.9 a	1.3	177.3 a	3.1 a
T^e^	T^e^ Amb	9.7	11.4 b	0.4	54.6 a	83.0	1.2	160.2	2.9 a
	T^e^ Amb+4°C	8.9	13.9 a	0.4	46.1 b	88.5	1.3	159.5	2.0 b
Irrigation	Control	10.2 a	12.1	0.4	53.4 a	79.4 b	1.1 b	157.7	2.2 b
	Drought	8.4 b	13.1	0.4	47.3 b	92.1 a	1.4 a	163.4	2.6 a
***Signification***									
CO_2_		**	**	**	***	*	ns	***	***
T^e^		ns	*	ns	**	ns	ns	ns	***
Irrigation		**	ns	ns	*	*	**	ns	*
CO_2_ × T^e^		ns	ns	ns	ns	ns	ns	ns	ns
CO_2_ × Irrigation		ns	ns	ns	ns	*	ns	ns	**
T^e^ × Irrigation		ns	ns	ns	ns	ns	ns	ns	ns
CO_2_ × T^e^ × Irrigation		ns	ns	ns	ns	ns	ns	ns	ns
				**B) Adesoto**					
**Principal Effects**									
CO_2_	CO_2_ Amb	9.1	15.9	1.0	39.0 b	91.1	1.4	157.2	2.6
	CO_2_ Elev	9.2	16.6	0.4	47.2 a	86.2	1.4	161.3	2.6
T^e^	T^e^ Amb	9.6	15.9	0.4	46.2	85.3	1.4	158.1	2.8
	T^e^ Amb+4°C	8.8	16.6	0.9	40.5	91.0	1.4	159.4	2.4
Irrigation	Control	9.2	17.7	0.4	51.5 a	89.3	1.2 b	169.3	1.9 b
	Drought	9.2	15.0	1.0	35.5 b	87.4	1.6 a	149.1	3.2 a
***Signification***									
CO_2_		ns	ns	ns	*	ns	ns	ns	ns
T^e^		ns	ns	ns	ns	ns	ns	ns	ns
Irrigation		ns	ns	ns	***	ns	***	ns	***
CO_2_ × T^e^		ns	ns	ns	ns	ns	ns	ns	ns
CO_2_ × Irrigation		ns	ns	ns	ns	ns	ns	ns	ns
T^e^ × Irrigation		ns	ns	ns	ns	ns	ns	ns	ns
CO_2_ × T^e^ × Irrigation		ns	ns	ns	*	*	ns	*	ns

Three-way ANOVA was performed for linear model, on raw data. Significance: *P ≤ 0.05, **P ≤ 0.01, ***P ≤ 0.001 and ns indicates not significant. Comparison means by Duncan's test (P ≤ 0.05) were shown for the significant interaction among treatments. Different letters indicate significant differences among data within the same factor. Amb, Ambient; Elev, Elevated; T^e^, Temperature.

### Correlations Between the Physiological Traits, Soluble Sugars, and Proline Content

Pearson correlation analysis was conducted between the physiological traits and content of biochemical compounds after 23 d of climate change-like conditions. Osmotic potential (Ψ_Π_) was negatively correlated with different sugar concentration depending on the tissue and genotype studied. In GF677–Catherina leaves, the content of sucrose, TSS, and proline was negatively correlated with osmotic potential. In the Adesoto–Catherina combination, the content of xylose in leaves, sorbitol and TSS in roots, and proline in both tissues showed negative correlation with osmotic potential. Photosynthetic rate in leaves of 'Catherina' grafted on GF677 and Adesoto were positively correlated with sucrose and proline. Positive correlations were also detected between WUE and content of sorbitol (0.521**, 0.534**), TSS (0.515**, 0.503**), and proline (0.461**, 0.474**) in leaves of GF677–Catherina and roots of Adesoto, respectively. Also in roots of plants grafted on GF677, WUE was positively correlated with TSS (0.543**) and proline (0.612***). The content of xylose in leaves of Adesoto–Catherina, and raffinose in roots of GF677, were negatively correlated with most of the physiological parameters except for WUE (**Table 4**).

**Table 4:** Pearson correlations between the physiological traits and biochemical content in leaves and roots of ‘Catherina’ plants grafted on GF677 and Adesoto rootstocks and subjected to climate change conditions for 23 days (n = 32).

Rootstock			Leaves				Rootstock			Roots			
GF677	Fructose	Sucrose	Xylose	Sorbitol	TSS	Proline	GF677	Fructose	Glucose	Raffinose	Sorbitol	TSS	Proline
Ψ_stem_	0.474^**^									-0.494^**^	0.524^**^		-0.585^***^
Ψπ		-0.483^**^			-0.507^**^	-0.666^***^							
RWC	0.507^***^	0.402^*^								-0.505^**^	0.547^***^		0.583^***^
*A*_N_	0.553^**^	0.679^***^				0.424^*^							-0.367^*^
*g*_s_				-0.501^**^					0.457^*^		0.431^*^		-0.394^*^
*E*				0.493^**^						-0.488^**^			0.584^***^
WUE				0.521^**^	0.515^**^	0.461^**^			0.727^***^		-0.379^*^	0.543^**^	0.612^***^
****													
**Adesoto**			**Leaves**				**Adesoto**			**Roots**			
Ψ_stem_		-0.716^***^	-0.642^***^			-0.533^**^		0.519^**^			-0.681^***^		
Ψπ			-0.460^**^			-0.391^*^					-0.589^***^	-0.507^**^	-0.507^**^
RWC													
*A*_N_		0.610^***^	-0.529^**^			0.586^***^		0.601^***^			-0.422^*^		
*g*_s_			-0.455^**^			-0.508^**^					-0.429^*^		-0.443^*^
*E*		0.576^***^	-0.549^***^			0.589^***^		0.504^**^			-0.447^*^		
WUE			0.449^*^								0.534^**^	0.503^**^	0.474^**^
													

Significance level: P ≤ 0.05 (*); P ≤ 0.01 (**) and P ≤ 0.001 (***); Ψstem, stem water potential; Ψπ, osmotic potential; RWC, relative water content; A_N_, photosynthesis; g_s_, conductance; E, transpiration; WUE, water use efficiency (A_N_ /g_s_), TSS, total soluble sugars.

### Effect of Climate Change on Transcriptional Responses

After 23 d of growth under climate change conditions, samples from roots and scion leaves of ‘Catherina’ budded on GF677 and Adesoto rootstocks were collected to study the transcriptomic responses. The transcript levels were evaluated by RT-qPCR for nine and seven genes, in roots and leaves, respectively. We focused on the significant changes under stress conditions concerning relative gene expression (RNorm) in both tissues and rootstocks (**Tables 5** and **6**).

**Table 5:** Gene expression (Rnorm values) in root tissue (n = 4) under ambient (amb CO_2_) and high (CO_2_ elev) CO_2_, ambient (T^e^ amb) and high (T^e^ amb + 4°C) temperature, and control irrigation and drought-stressed GF677 (A) and Adesoto (B) *Prunus* rootstocks budded with cv. Catherina, after 23 days of treatment.

Roots		*SDH*	*S6PDH*	*SIP1*	*P5CS*	*P5CR*	*PIP2*	*DREB2*	*AREB2*	*HAT22*
A) GF677										
**Principal Effects**										
CO_2_	CO_2_ Amb	9.5	0.03	1.1 a	0.5	1.8	0.1	0.4	0.2	1.4
	CO_2_ Elev	9.1	0.01	0.9 b	0.7	2.1	0.1	0.3	0.2	1.7
T^e^	T^e^ Amb	13.3 a	0.03 a	1.2	0.7 a	2.2	0.1	0.5 a	0.2 a	2.1 a
	T^e^ Amb+4°C	5.3 b	0.01 b	0.7	0.5 b	1.7	0.1	0.2 b	0.1 b	1.0 b
Irrigation	Control	14.5 a	0.01 b	0.6	0.3 b	1.6 b	0.1	0.4	0.2	1.7
	Drought	4.02 b	0.03 a	1.4	0.9 a	2.3 a	0.1	0.4	0.2	1.3
***Signification***										
CO_2_		ns	ns	*	ns	ns	ns	ns	ns	ns
T^e^		***	**	ns	*	ns	ns	*	***	***
Irrigation		***	**	ns	***	*	ns	ns	ns	ns
CO_2_ × T^e^		ns	*	ns	ns	ns	ns	ns	ns	ns
CO_2_ × Irrigation		ns	ns	*	ns	ns	ns	ns	*	ns
T^e^ × Irrigation		***	*	ns	ns	ns	ns	ns	ns	ns
CO_2_ × T^e^ × Irrigation		ns	ns	ns	ns	ns	ns	ns	ns	ns
**B) Adesoto**										
**Principal Effects**										
CO_2_	CO_2_ Amb	7.8	0.004 b	0.5	0.4	1.2	0.11	0.6	0.14 b	1.3
	CO_2_ Elev	10.8	0.034 a	0.9	0.8	1.9	0.11	0.7	0.22 a	1.8
T^e^	T^e^ Amb	10.0	0.005	0.7	0.5	1.4	0.12	0.8	0.17	1.6
	T^e^ Amb+4°C	8.6	0.032	0.7	0.7	1.8	0.10	0.5	0.19	1.5
Irrigation	Control	12.8 a	0.006	0.6	0.4	1.6	0.12	0.6	0.18	1.2
	Drought	5.8 b	0.032	0.9	0.7	1.5	0.10	0.6	0.17	1.9
***Signification***										
CO_2_		ns	**	ns	ns	ns	ns	ns	*	ns
T^e^		ns	ns	ns	ns	ns	ns	ns	ns	ns
Irrigation		**	ns	ns	ns	ns	ns	ns	ns	ns
CO_2_ × T^e^		ns	ns	ns	ns	ns	ns	ns	ns	ns
CO_2_ × Irrigation		ns	ns	ns	ns	ns	**	ns	ns	ns
T^e^ × Irrigation		ns	ns	ns	ns	ns	ns	ns	ns	ns
CO_2_ × T^e^ × Irrigation		ns	*	ns	ns	ns	ns	ns	ns	ns

Three-way ANOVA was performed for lineal model on raw data. Significance: *P ≤ 0.05, **P ≤ 0.01, ***P ≤ 0.001 and ns indicates not significant. Comparison means by Duncan's test (P ≤ 0.05) were shown for the significant interaction among treatments. Different letters indicate significant differences among data within the same factor. Amb, Ambient; Elev, Elevated; T^e^, Temperature.

**Table 6:** Gene expression (Rnorm values) in leaf tissue (n = 4) under ambient (amb CO_2_) and high (CO_2_ elev) CO_2_, ambient (T^e^ amb) and high (T^e^ amb + 4°C) temperature, and control irrigation and drought-stressed GF677 (A) and Adesoto (B) *Prunus* rootstocks budded with cv. Catherina, after 23 days of treatment.

Leaves		*SDH*	*S6PDH*	*SIP1*	*P5CS*	*P5CR*	*OAT*	*HAT22*
A) GF677								
**Principal Effects**								
CO_2_	CO_2_ Amb	0.3	161.3	5.2	5.5	10.3	32.2	8.3 a
	CO_2_ Elev	0.1	178.2	3.0	4.7	5.8	24.3	5.0 b
T^e^	T^e^ Amb	0.3	219.0	3.9	5.5	6.8	25.5	6.9
	T^e^ Amb+4°C	0.1	120.5	4.4	4.7	9.3	30.9	6.4
Irrigation	Control	0.2	106.7 b	2.4 b	4.6	10.3	24.6	5.2 b
	Drought	0.2	232.8 a	5.8 a	5.7	5.9	31.9	8.2 a
***Signification***								
CO_2_		ns	ns	ns	ns	ns	ns	**
T^e^		ns	ns	ns	ns	ns	ns	ns
Irrigation		ns	*	**	ns	ns	ns	*
CO_2_ × T^e^		ns	ns	*	*	**	ns	ns
CO_2_ × Irrigation		ns	ns	ns	ns	*	ns	*
T^e^ × Irrigation		ns	ns	ns	ns	*	ns	ns
CO_2_ × T^e^ × Irrigation		ns	ns	*	ns	ns	ns	ns
**B) Adesoto**								
**Principal Effects**								
CO_2_	CO_2_ Amb	0.009 b	44.1	0.8	0.9	1.9 a	9.8	2.6 a
	CO_2_ Elev	0.036 a	34.3	0.5	0.8	1.2 b	7.7	1.6 b
T^e^	T^e^ Amb	0.009 b	46.9	0.7	0.8	1.5	8.1	1.9
	T^e^ Amb+4°C	0.036 a	31.5	0.6	0.9	1.7	9.3	2.3
Irrigation	Control	0.027	31.7	0.5	0.9	2.2 a	9.0	1.9
	Drought	0.018	46.7	0.9	0.7	1.0 b	8.4	2.3
***Signification***								
CO_2_		*	ns	ns	ns	*	ns	*
T^e^		*	ns	ns	ns	ns	ns	ns
Irrigation		ns	ns	ns	ns	**	ns	ns
CO_2_ × T^e^		*	ns	ns	*	ns	ns	ns
CO_2_ × Irrigation		ns	ns	ns	ns	ns	ns	ns
T^e^ × Irrigation		ns	ns	ns	ns	*	ns	ns
CO_2_ × T^e^ × Irrigation		ns	ns	ns	ns	*	*	ns

Three-way ANOVA was performed for lineal model on raw data. Significance: *P ≤ 0.05, **P ≤ 0.01 and ns indicates not significant. Comparison means by Duncan's test (*P*≤0.05) were shown for the significant interaction among treatments. Different letters indicate significant differences among data within the same factor. Amb, Ambient, Elev, Elevated; T^e^, Temperature.

For roots of GF677 rootstock (**Table 5A**), we found that the CO_2_ treatment significantly decreased the transcript level of raffinose synthase (*SIP1*), which encodes an enzyme involved in raffinose biosynthesis. The temperature treatment strongly affected the expression of genes related to sugar and proline metabolism, as well as other genes associated with the drought stress responses. We observed decrease expression of sorbitol dehydrogenase (*SDH*) and sorbitol-6-phosphate dehydrogenase (*S6PDH*), key enzymes of sorbitol catabolism and biosynthesis, respectively. Elevated temperature also decreased the expression of Δ-1-pyrrolyne-carboxylate synthase (*P5CS*), dehydration responsive element binding protein 2 (*DREB2*), ABA responsive element binding protein (*AREB2*), and homeodomain-leucine zipper protein (*HAT22*). Finally, water deficit in roots of GF677 also diminished the expression of *SDH* while the normalized expression of *S6PDH,* Δ-1-pyrrolyne-carboxylate synthase (*P5CS*), and Δ-1-pyrrolyne-carboxylate reductase (*P5CR*) was upregulated (**Table 5A**). These results were consistent with the increase in proline content in roots of GF677 under drought stress (**Table 2A**). In roots of the Adesoto rootstock, only four genes were affected by the climate change-like conditions (**Table 5B**). Elevated CO_2_ significantly increased the transcript levels of *S6PDH* and *AREB2*, while drought condition downregulated the expression of *SDH*. The interactive effect of CO_2_ and irrigation modified *PIP2* gene expression, but without a clear trend (**Supplementary Tables 6**and **7**). It is important to note that under drought stress, gene regulation of sorbitol metabolism (downregulated catabolism) was consistent with the increase in sorbitol content in roots of Adesoto (**Table 2B**).

Our results showed that in ‘Catherina' scion leaves, elevated CO_2_ downregulated *HAT22* when budded either on GF677 or Adesoto (**Table 6**). When 'Catherina' was grafted on GF677, drought stress significantly enhanced the expression of *S6PDH, SIP1*, and *HAT22* (**Table 6A**, **Supplementary Table 8**). When 'Catherina' was grafted on Adesoto, elevated CO_2_ and temperature, as well as its interaction, significantly enhanced the expression of *SDH* in scion leaves (**Table 6B**, **Supplementary Table 9**). The transcript level of *P5CR* decreased in the same rootstock with elevated CO_2_ and drought stress treatments, but the double and triple interactions did not follow the same trend (**Table 6B**, **Supplementary Table 9**). Regarding the ornithine aminotransferase (*OAT*) gene, which encodes an enzyme that synthesizes a precursor for proline biosynthesis, significant differences in its expression were found only when plants were grafted on Adesoto in the triple interaction, but without a clear trend (**Supplementary Table 9**). Interestingly, under drought stress conditions, gene regulation related to sorbitol metabolism was consistent with the increase in sorbitol content in scion leaves of GF677 (**Table 3A**). Furthermore, the lack of accumulation of sorbitol in scion leaves of Adesoto under elevated CO_2_ and temperature may be due to the upregulation of its catabolism (*SDH*).

In summary, gene regulation under climate change conditions was divers and depended on the stress, tissue, and genotype (**Supplementary Table 10**). Concerning tissues, in roots nine different genes associated with specific treatments were modified; eight were affected in GF677, and four in Adesoto. In leaves, five genes were differently expressed on cv. Catherina budded on each rootstock, GF677 or Adesoto. Concerning genotypes, GF677 rootstock showed differences in the expression of 13 genes in both tissues, with roots (eight genes) being more affected than scion leaves (five genes). Adesoto rootstock was less affected and only nine genes were modified, four genes in roots and five in scion leaves, respectively. Finally, concerning stresses, in plants grafted on GF677 rootstock, elevated CO_2_ significantly modified the expression of only two genes, one in each organ tissue, while elevated temperature affected six genes (all in roots), and as a response to the irrigation treatment, seven genes (four in roots, three in leaves) were differentially expressed. Concerning plants grafted on Adesoto rootstock, CO_2_ treatment altered the expression of five genes (two in roots and three in scion leaves), while elevated temperature (in scion leaves) and irrigation (in roots and scion leaves) modified the expression of only one gene. Elevated temperature did not affect gene expression in leaves of 'Catherina' grafted on GF677 or roots of Adesoto rootstock. The triple interaction between CO_2_, temperature, and irrigation affected only one gene (*SIP1*) in 'Catherina' grafted on GF677 rootstock (in leaf tissue) and three genes in the Adesoto–Catherina combination (*S6PDH* in roots, and *P5CR* and *OAT* in scion leaves). The double interaction between treatments involved transcriptome variations mainly in leaf tissues.

## Discussion

### Effect of Climate Change-Like Conditions (Elevated CO_2_ and Temperature, and Low Irrigation) on Growth and Physiological Status

The decrease in shoot/root DW ratio under elevated CO_2_ and drought suggests that root growth is more stimulated than the aerial part, which was also reported in other plant species (Madhu and Hatfeld, 2013; Medina et al., 2016), although these changes depend on interactions between genotype and environment (Medina et al., 2016). Temperature did not affect biomass as was reported in other plant species (Gray and Brady, 2016). Drought decreased leaf DW and shoot/root ratio in both genotypes, which is in accordance with previous studies in grapevine and wheat (Kizildeniz et al., 2015; Medina et al., 2016) and in *Prunus* genotypes (Jiménez et al., 2013). Under water deficit, investment in root growth over leaf growth has the benefit of reducing the aerial part avoiding water loss *via* transpiration (Gray and Brady, 2016). This effect was more evident in plants grafted on the GF677 rootstock that also had increased SPAD values as a consequence of the chlorophyll accumulation. Elevated CO_2_ alone or in combination with elevated temperature attenuated the negative effect of drought on plant growth in both rootstocks (Fattahi et al., 2019), as was reported previously for elevated CO_2_ in drought-stressed grapevine plants (Kizildeniz et al., 2015) and in waterlogged cherry rootstocks (Pérez-Jiménez et al., 2018). In the future, climate change conditions may alleviate drought effects in *Prunus* species, as has been reported for other trees (Kelly et al., 2016).

The photosynthetic rates of plants grafted on GF677 were higher with elevated CO_2_, but lower with elevated temperature and drought stress. However, the photosynthetic rates of plants grafted on Adesoto decreased only under drought stress conditions. Previous work has shown that elevated CO_2_ can lead to increases in photosynthetic rates in some plant species, but often involve acclimation process that limit yield and production of biomass. Under such conditions, we found in plants grafted on GF677 an increase in photosynthesis and biomass, while those grafted on Adesoto showed not significant changes. After 23 d of exposure to elevated CO_2_, plants grafted on Adesoto rootstock showed acclimation effects irrespective of the temperature and drought stress conditions, as shown in grapevines (Leibar et al., 2015). Leakey et al. (2009) described genetic factors that predispose plants to a greater acclimation of photosynthesis and mentioned the importance of an unbalance in sink capacity leading to increases in foliar carbohydrates. Apparently, this is not the case in the *Prunus* rootstocks studied herein, because photosynthetic acclimation was not associated with carbohydrate accumulation in scion leaves of GF677 or Adesoto (see sugars leaf/root ratio as fold change in stress compare to control, **Figure S1**). Differences expressed in a logarithmic basis often allow for easier comparison of preferential compound accumulation in leaves or roots. A positive value [Log_2_ (Stress/Control) > 1] indicates a preferential accumulation in scion leaves under stress conditions. On the contrary, a negative value [Log_2_ (Stress/Control) < 1] indicates preferential accumulation in roots under stress conditions. We found that in GF677–Catherina plants, elevated CO_2_ led to increased leaf biomass, decreased SLA, and accumulation of carbohydrates in roots and scion leaves did not provoke acclimation. Similarly, a lack of such acclimation was found in other species as *Populu*s trees that export photosynthates during the day and accumulate the overflow as starch to avoid acclimation, which acts to maintain *A*_N_ at elevated CO_2_ (Scarascia-Mugnozza et al., 2006).

High temperature and drought are frequently co-occurring stresses and they have a substantial impact on the performance and vitality of plants (Afzal et al., 2018). However, at the physiological level, temperature only affected plants grafted on GF677. In these conditions, plants of GF677 showed significantly decreased photosynthesis rates, reduced stomatal conductance, and decreased transpirational water loss, compared to control plants, as reported for grapevine (Leibar et al., 2015). Under drought stress conditions plants grafted on *Prunus* rootstocks showed a significant decrease in stem and osmotic potentials, photosynthetic rate (*A*_N_), stomatal conductance (*g*_s_), and transpiration rate, which is in agreement with previous studies (Mellisho et al., 2011; Jiménez et al., 2013; Pedroso et al., 2014; Ksouri et al., 2016; Haider et al., 2018). Stomatal closure is one of the earliest responses to water deficiency adopted by plants as a water saving strategy (Long et al., 2006; Serra et al., 2014; Nakhforoosh et al., 2015; Vicente et al., 2015; Pazzagli et al., 2016) to decrease evaporative water loss and maintain a water balance.

In plants grafted on both rootstocks, the combination of climate change-like stress conditions improved water-use efficiency (*A*_N_ *_/_g*_s_) (**Figure 2**) except for temperature in Adesoto–Catherina. Also, in both genotypes, the increase in atmospheric CO_2_ concentration, alone or combined with drought, decreased stomatal conductance and resulted in increases in WUE, as reported for grapevine (Leibar et al., 2015). On the contrary, Centritto and coworkers (Centritto et al., 1999) observed neither reduction of stomatal conductance nor changes in WUE in response to elevated CO_2_ in droughted cherry plantlets. In this study, elevated CO_2_ ameliorated the drought-induced decrease in photosynthesis only in plants grafted on GF677.

### Metabolic Rearrangements and Transcriptional Regulation in Response to Climate Change-Like Conditions (Elevated CO_2_ and Temperature, and Low Irrigation)

Plant growth depends on assimilation of carbohydrates, which are accumulated and mobilized in the form of soluble sugars under stress conditions (Fabbrin et al., 2015; Sami et al., 2016). In this experiment, changes in sugar content in scion leaves and roots under climate stress conditions are summarized as fold changes [FC = log(2) (Stress/control)] in **Figure S2**. Changes (increase or decrease are positive or negative values, respectively) were different between genotypes and among treatments and are related to the biomass changes found in each genotype and tissue combination. The increase in total sugars in roots and leaves in plants grafted on GF677 was consistent with the increase in dry weight in both tissues under elevated CO_2_ (**Figure S2**, **Table 1**). In the same way, increases in fructose, glucose, and total sugars in roots of Adesoto were consistent with the increase in root growth (decrease of shoot/root DW ratio, **Table 1**). It has been established that metabolic adjustments in response to unfavorable conditions are dynamic and multifaceted and not only depend on the type and strength of the stress, but also on the cultivar and the plant species (Krasensky and Jonak, 2012). In this study, elevated CO_2_ led to an increase in sugars in scion leaves and in roots in GF677 (**Figure S2**). However, only fructose, raffinose, sucrose, and sorbitol accumulated (**Figure S1**) more in scion leaves than in roots. Interestingly, in Adesoto soluble sugar concentration, except for sucrose, increased mainly in roots (**Figure S2**) and accumulated there (**Figure S1**) being this tissue the main sink. In both genotypes, under elevated CO_2_, independently of acclimation, the increase and accumulation of sucrose in leaves did not limit photosynthesis neither in plants grafted on GF677 nor Adesoto, which was supported by the positive correlation between sucrose and *A*_N_. Furthermore, the increase in proline and its accumulation in leaves of GF677–Catherina plants under elevated CO_2_ may be linked to the role of proline as a ROS scavenger protecting the photosynthetic apparatus from oxidative damage.

Accumulation of particular osmolytes (soluble sugars and/or proline) has been observed in different plant species under stress conditions (Krasensky and Jonak, 2012; Baslam et al., 2014; Fabbrin et al., 2015; Yu et al., 2015; Haider et al., 2017; Pérez-Jiménez et al., 2018) and is thought to help maintain osmolarity. Higher accumulation of compatible solutes may contribute to drought tolerance by protecting the photosynthetic apparatus (Krasensky and Jonak, 2012; Jiménez et al., 2013) and maintaining osmotic homeostasis (Jiménez et al., 2013). In particular, the increases in sorbitol in leaves, raffinose in roots, and proline in both tissues were related to a decrease in osmotic potential and an increase in WUE in *Prunus* rootstocks (Jiménez et al., 2013). In this study, comparable results were found in GF677 grown under drought stress (increases in raffinose and proline in roots, and sorbitol, xylose, and proline in scion leaves). In the GF677 rootstock under drought stress, sugars accumulated in the same organs, but proline was allocated preferentially in roots. In Adesoto, sorbitol, xylose, and proline increased (**Figure S2**) and accumulated (**Figure S1**) under drought stress condition in the same organs, in roots (sorbitol) and in scion leaves (xylose and proline). These changes were also consistent with the negative correlations found between the content of sugar and proline in scion leaves and roots versus stem water and osmotic potentials, indicating a role in maintaining water status in plants grafted on GF677 and Adesoto rootstocks (**Table 4**). We suggest that the accumulation of sorbitol, xylose, and proline in different plant tissues may increase the tolerance of *Prunus* trees to progressive drought stress (**Figure S1**). According to our results, we suggest that proline may act as an osmolyte in roots of GF677 and as a ROS scavenger in leaves of Adesoto–Catherina (partially in GF677) to protect each from oxidative damage (Krasensky and Jonak, 2012). In this regard, each genotype has the capacity to accumulate active solutes as osmolytes and the ability to maintain its own strategy to increase WUE.

Sorbitol is a major end product of photosynthesis that under moderate drought conditions, is preferentially synthesized over sucrose (Escobar-Gutiérrez et al., 1998), which is in agreement with the significant increases in sorbitol content we observed in scion leaves and roots and the decreased sucrose levels in scion leaves in the drought-tolerant GF677. On the contrary, in Adesoto, another pattern was found for sorbitol, which increased only in roots and preferentially accumulated along with sucrose in roots (**Figures S1** and **S2**). Several studies on *Prunus* rootstocks confirmed that sorbitol content in scion leaves (Ranney et al., 1991; Haider et al., 2018) and in roots (Ranney et al., 1991) were enhanced resulting in active osmotic adjustment and decreased osmotic potential, which increased plant resistance to drought stress (Arndt et al., 2000; Krasensky and Jonak, 2012). Apparently, scion leaf sorbitol in GF677–Catherina did not result in osmotic adjustment, while root sorbitol in Adesoto rootstock negatively correlated with osmotic potential (**Table 4**). Functions of sorbitol—other than osmotic adjustment—such as translocation and storage of carbon, cryoprotection, and prevention of reactive oxygen species, have been described previously (Lo Bianco et al., 2000). The osmotic adjustment of sorbitol in roots may influence shoot/root partitioning patterns and root growth, and may indirectly control plant growth in responses to water deficit (Turner, 1986). We may speculate that the allocation of sorbitol in roots of Adesoto may be a stimulus to increase the photosynthetic rate and evade or reduce the acclimation process.

In order to shed light on the complex regulatory networks of all changes at the molecular level caused by climate change-like stress conditions, we followed the expression of genes that regulate sugar and proline metabolism. Sorbitol levels were determined by the balance between biosynthesis and catabolism. Sorbitol is synthesized by S6PDH (sorbitol-6-phosphate dehydrogenase) in source leaves, translocated through phloem, and catabolized by SDH in fruit (Suzuki and Dandekar, 2014) and other sink organs. As a result of drought stress, the increase in sorbitol content in scion leaves of GF677 was consistent with the upregulation of *S6PDH* as found previously in peach leaves (Sakanishi et al., 1998). However under climate change-like stress conditions, *SDH* transcript levels were not correlated with sorbitol content, suggesting that other factors have significant regulatory effects, as other authors have pointed out under control conditions (Wu et al., 2010). Likewise, the enhanced expression of *SIP1* in scion leaves of GF677 under drought stress did not provide evidence supporting a role of raffinose in stress tolerance as was reported in *Prunus* rootstocks (Jiménez et al., 2013) or in Arabidopsis plants (Krasensky and Jonak, 2012).

Proline is biosynthesized in plants through two successive reductions catalyzed by 1-pyrroline-5-carboxylate synthetase and pyrroline-5-carboxylate reductase (Verbruggen and Hermans, 2008). In the present study, the significant increases in *P5CS* and *P5CR* under drought stress were accompanied by higher proline content in roots of the tolerant rootstock GF677 as it was found in *P5CS* in GF677 and Cadaman rootstocks (Jiménez et al., 2013). However, in leaves of 'Catherina' grafted on Adesoto, we found the opposite. *P5CR* was downregulated while proline content increased under drought stress without evidences that confirmed the synthesis of proline precursor through the ornithine alternative pathway (Miller et al., 2009). The apparent inconsistency for the downregulation of *P5CR* under elevated CO_2_ and drought treatments and the increase of proline in scion leaves of Adesoto could be explained by gene regulation linked to the interaction. In line with the contrasting results found in both genotypes in this study, Szabados and Savoure (2010) reported that the correlation between proline content and abiotic stress in plants is not always positive and may be genotype-dependent.

Finally, to better establish differences among stresses and to understand the regulation of the physiological and molecular responses found among the genotypes, we explored gene expression of stress-inducible and -responsive genes. It has been described that many stress-inducible genes are enriched in motifs that are binding targets of transcription factors (drought or ABA-regulated genes) (Huang et al., 2008). Numerous gene families and transcription factors (TFs) are implicated in the defense responses to stress in plants through regulation of metabolites levels. *DREB2*, which encodes a DRE/CRT-binding protein, activates the expression of genes related to osmoprotectant and antioxidant biosynthesis and whose expression is rapidly induced by osmotic stress (Song et al., 2014). However, our results are in contrast with the upregulation found under high temperature in poplar (Song et al., 2014) and under drought stress in peach leaves (Haider et al., 2018). In our experimental conditions, the lack of activation of *DREB2* could be due to elevated temperature or drought stress in *Prunus* rootstocks not related to oxidative damage as found in Arabidopsis (Hwang et al., 2012). Furthermore, *AREB* encodes a major TF involved in abiotic stress responses in Arabidopsis (Fujita et al., 2013; Nakashima et al., 2014). Overexpression of *AREB1* in rice and soybean improved drought tolerance (Oh et al., 2005), while overexpression of *AREB2* in apple led to increased sugar accumulation (Ma et al., 2017). In our study, the downregulation of *AREB* in roots of GF677 under elevated temperature may be linked to the lack of significant accumulation of sugars in this organ. On the contrary, upregulation of *AREB* in roots of Adesoto under elevated CO_2_ treatment may be linked to the accumulation of total sugars (**Figure S1**). Finally, the homeodomain-leucine zipper (HD-ZPII) gene (*HAT22)* was upregulated in scion leaves of GF677 under drought stress conditions, as was found previously in cotton plants as a response to drought stress (Hou et al., 2018) and in Arabidopsis in response to ABA treatment and drought (Liu et al., 2016). It has been reported that the dehydration-responsive homeodomain-leucine zipper gene family (HD-Zips) show modulated expression in response to dehydration in leaves and roots (Deng et al., 2002), supporting the role of HD-Zips in regulatory pathways that lead to desiccation tolerance. Results found in plants grafted on GF677 may be in agreement with the model proposed in Arabidopsis (Liu et al., 2016) concerning the role of *HAT22/ABIG1* TF. In this combination, drought may act through ABA to increase *HAT22* transcription to limit shoot growth and promote leaf senescence. In contrast, in Adesoto–Catherina the response concerning shoot growth and senescence was less evident.

Taken into account all these transcriptomic changes, it underscores the difficulty in understanding the global context of multi-stress responses. The broad number of genes that are differentially modified under environmental stress conditions reveals the complex regulatory network of TFs controlling plant responses at the morphological, physiological, and molecular level.

## Conclusion

Climate change will alter future plant growth conditions and, in this scenario, knowledge of the plasticity of *Prunus* rootstocks will be critical for peach production. Elevated CO_2_, elevated temperature, and drought stress were applied to simulate future climate conditions and to compare two contrasting *Prunus* rootstocks for ‘Catherina' peach at the physiological, molecular, and transcriptomic level. This study revealed that the impact of climate change was not uniform for *Prunus* species and the responses depend on the genetic background and the performance of the genotypes facing the stress in a specific manner.

In response to stress, morphological and physiological changes were accompanied by molecular and transcriptomic changes in a coordinated manner, but depending on the rootstock. Elevated CO_2_ increased photosynthetic rates in plants grafted on GF677, while in plants grafted in Adesoto, acclimation was observed. At the molecular level, metabolite content was affected by climate change-like stress factors such that soluble sugars and proline were partitioned in different shoot:root patterns depending on the stress and the genotype. Under elevated CO_2_, osmoprotectants accumulated in leaves of GF677–Catherina, while in Adesoto–Catherina these metabolites accumulated mainly in roots. The metabolic adjustments developed in response to stress involved pathways controlling levels of sugar and proline that were highly coordinated and regulated at the transcriptomic level (*SDH*, *S6PDH*, *SIP1*, *P5CR*, and *P5CS*) in both tissues and genotypes.

Stress tolerance is a complex trait that is controlled by multiple genes. GF677–grafted plants showed more changes than those grafted on Adesoto, scion leaves were more affected than roots, although some responses were quite similar for both genotypes and varied depending on the stress and the affected tissue. We conclude that both peach rootstocks may be tolerant to climate change, but the strategies employed by each genotype in response to stress are different and are associated with the genetic background.

GF677 is a tolerant rootstock that utilizes a range of machinery to maintain good performance, and control plant growth and senescence under stress. At elevated CO_2_, plants increase *A*_N_, and as a consequence, the rootstock needs to control oxidative stress and plant growth. This genotype increased proline content in scion leaves as a ROS scavenger and downregulated *HAT22* to avoid senescence and increase leaf growth, to create a better balance with root growth. At elevated temperature, no significant changes were found in growth, but all transcriptomic changes were in roots to control root growth and senescence *via* downregulation of genes (*AREB2* to reduce the accumulation of sugars and *HAT22* to restrict growth and senescence). Under drought stress, this rootstock controls leaf growth *via* the upregulation of *HAT22*, which may result in a decrease in aerial growth in favor of root growth to improve water uptake. Adesoto is a resilient rootstock suitable to grow under climate change stress conditions. Under elevated CO_2_, this rootstock is able to control photosynthesis, growth, and sugar biosynthesis. It is also insensitive to elevated temperature, and under drought stress, maintains water status through metabolic balance among tissues. In scion leaves, *HAT22* was downregulated maintaining growth of the aerial part. In roots, *AREB2* was upregulated to promote accumulation of sugars and indirectly stimulate photosynthesis. This study confirms the importance of rootstocks in sensing stress, regulating of scion growth, and conferring tolerance to the variety. This work establishes a basis for developing screening methods that may enable early selection of woody tree species adapted to new environmental scenarios.

## Data Availability Statement

All datasets generated for this study are included in the article/**Supplementary Material**.

## Author Contributions

SJ and YG devised the study objectives and designed the experiment. SJ carried out the experiment, conducted the statistical analysis and drafted the manuscript. MF contributed to the writing of the paper, to the statistical analysis and figure preparation. KB conducted the expression analysis work. SN-m contributed to the writing. JJI helped with the experimental facilities and physiological measures and supervised the manuscript. YG conceived the experiment, wrote and edited the paper and helped with the statistical analysis. All authors read and approved the manuscript.

## Funding

This work was partly funded by the Spanish Ministry of Economy and Competitiveness grants AGL2014-52063R, AGL2017-83358-R (MCIU/AEI/FEDER/UE); and the Government of Aragón with grants A44, A09_17R and La Caixa-GA-0007/2010 which were co-financed with FEDER funds. MF and SN-M were recipients of Iranian fellowships. SJ was supported by JAE-Doc-CSIC contract co-funded by ESF. KB received a Master's fellowship awarded by the CIHEAM-IAMZ.

## Conflict of Interest

Author SJ was employed by company Bayer AG, though the research was conducted before his employment at the company, in the absence of any conflict of interest.

The remaining authors declare that the research was conducted in the absence of any commercial or financial relationships that could be construed as a potential conflict of interest.

## References

[B1] Ábrahám E. Hourton-Cabassa C. Erdei L. Szabados L. (2010). Methods for determination of proline in plants. Methods Mol. Biol. 639, 317–331. 10.1007/978-1-60761-702-0_20 20387056

[B2] Afzal M. Shabbir G. Ilyas M. Jan S.S.A. Jan S. A. (2018). Impact of climate change on crop adaptation: current challenges and future perspectives. Pure Appl. Biol. 7, 965–972. 10.19045/bspab.2018.700115

[B3] Ainsworth E. A. Leakey A. D. B. Ort D. R. Long S. P. (2008). FACE-ing the facts: inconsistencies and interdependence among field, chamber and modeling studies of elevated CO_2_ impacts on crop yield and food supply. New Phytol. 179, 5–9. 10.1111/j.1469-8137.2008.02500.x 18482226

[B4] Aranjuelo I. Irigoyen J. J. Perez P. Martinez-Carrasco R. Sanchez-Díaz M. (2005). The use of temperature gradient tunnels for studying the combined effect of CO_2_, temperature and water availability in N_2_ fixing alfalfa plants. Ann. Appl. Biol. 146, 51–60. 10.1111/j.1744-7348.2005.04074.x

[B5] Aranjuelo I. Irigoyen J. J. Perez P. Martinez-Carrasco R. Sanchez-Diaz M. (2006). Response of nodulated alfalfa to water supply, temperature and elevated CO_2_: Productivity and water relations. Environ. Exp. Bot. 55, 130–141. 10.1016/j.envexpbot.2004.10.007

[B6] Aranjuelo I. Cabrera-Bosquet L. Morcuende R. Avice J. C. Nogués S. Araus J. L. (2011). Does ear C sink strength contribute to overcoming photosynthetic acclimation of wheat plants exposed to elevated CO_2_? J. Exp. Bot. 62, 3957–3969. 10.1093/jxb/err095 21511906 PMC3134354

[B7] Arndt S. K. Wanek W. Clifford S. C. Popp M. (2000). Contrasting adaptations to drought stress in field-grown *Ziziphus mauritiana* and *Prunus persica* trees: water relations, osmotic adjustment and carbon isotope composition. Aust. J. Grape Wine Res. 27, 985–996. 10.1071/PP00022

[B8] Arp W. (1991). Effects of source–sink relations on photosynthetic acclimation to elevated CO_2_ . Plant Cell Environ. 14, 869–875. 10.1111/j.1365-3040.1991.tb01450.x

[B9] Baslam M. Antolín M. C. Gogorcena Y. Muñoz F. Goicoechea N. (2014). Changes in alfalfa forage quality and stem carbohydrates induced by arbuscular mycorrhizal fungi and elevated atmospheric CO_2_ . Ann. Appl. Biol. 164, 190–199. 10.1111/aab.12092

[B10] Bates L. S. Waldren R. P. Teare I. D. (1973). Rapid determination of free proline for water-stress studies. Plant Soil 39, 205–207. 10.1007/BF00018060

[B11] Bencze S. Bamberger Z. Janda T. Balla K. Varga B. Bedö Z. (2014). Physiological response of wheat varieties to elevated atmospheric CO_2_ and low water supply levels. Photosynthetica 52, 71–82. 10.1007/s11099-014-0008-y

[B12] Cattivelli L. Rizza F. Badeck F. Mazzucotelli E. Mastrangelo A. M. Francia E. (2008). Drought tolerance improvement in crop plants : an integrated view from breeding to genomics. Field Crops Res. 105, 1–14. 10.1016/j.fcr.2007.07.004

[B13] Centritto M. Lee H. Jarvis P. (1999). Interactive effects of elevated [CO_2_] and drought on cherry (*Prunus avium*) seedlings. New Phytol. 141, 129–140. 10.1046/j.1469-8137.1999.00327.x

[B14] Deng X. Phillips J. Meijer A. H. Salamini F. Bartels D. (2002). Characterization of five novel dehydration-responsive homeodomain leucine zipper genes from the resurrection plant *Craterostigma plantagineum* . Plant Mol. Biol. 49, 601–610. 10.1023/A:1015501205303 12081368

[B15] Erice G. Sanz-Sáez A. Urdiain A. Araus J. L. Irigoyen J. J. Aranjuelo I. (2014). Harvest index combined with impaired N availability constrains the responsiveness of durum wheat to elevated CO_2_ concentration and terminal water stress. Funct. Plant Biol. 41, 1138–1147. 10.1071/FP14045 32481064

[B16] Escobar-Gutiérrez A. Zipperlin B. Carbonne F. Moing A. Gaudillere J. (1998). Photosynthesis, carbon partitioning and metabolite content during drought stress in peach seedlings. Aust. J. Plant Physiol. 25, 197–205. 10.1071/PP97121

[B17] Fabbrin E. G. Gogorcena Y. Mogor A. F. Garmendia I. Goicoechea N. (2015). Pearl millet growth and biochemical alterations determined by mycorrhizal inoculation, water availability and atmospheric CO_2_ concentration. Crop Pasture Sci. 66, 831–840. 10.1071/CP14089

[B18] FAO (2016). The State of Food and Agriculture: Climate Change, Agriculture and Food Security (Rome: Food and Agriculture Organization of the United Nations).

[B19] Fathi H. Amiri M. E. Imani A. Hajilou J. Nikbakht J. (2017). Response of almond genotypes/cultivars grafted on GN15 “Garnem” rootstock in deficit-irrigation stress conditions. J. Nuts 8, 123–135. 10.22034/jon.2017.536243

[B20] Fattahi M. Jiménez S. Nasrolahpour-moghadam S. Irigoyen J. Gogorcena Y. (2019). “Effects of climate change on peach grafted into two contrasting *Prunus* spp. rootstocks,” in XXIII of the Spanish Society of Plant Physiology (Pamplona (Spain): Spanish Society of Plant Physiology), 89.

[B21] Fujita Y. Yoshida T. Yamaguchi-Shinozaki K. (2013). Pivotal role of the AREB/ABF-SnRK2 pathway in ABRE-mediated transcription in response to osmotic stress in plants. Physiol. Plant 147, 15–27. 10.1111/j.1399-3054.2012.01635.x 22519646

[B22] García-Sánchez F. Syvertsen J. P. Gimeno V. Botía P. Perez-Perez J. G. (2007). Responses to flooding and drought stress by two citrus rootstock seedlings with different water-use efficiency. Physiol. Plant 130, 532–542. 10.1111/j.1399-3054.2007.00925.x

[B23] Gogorcena Y. Iturbe-Ormaetxe I. Escuredo P. R. Becana M. (1995). Antioxidant defenses against activated oxygen in pea nodules subjected to water stress. Plant Physiol. 108, 753–759. 10.1104/pp.108.2.753 12228507 PMC157397

[B24] Gogorcena Y. Sánchez G. Moreno S. Pérez S. Ksouri N. (2020). “Genomic-based breeding for climate-smart peach varieties,” in Genome designing of climate fruit crops. Ed. Kole Ch. (Cham, Switzerland AG: Springer Nature). 291–351. 10.1007/978-3-319-97946-5_9

[B25] Gray S. B. Brady S. M. (2016). Plant developmental responses to climate change. Dev. Biol. 419, 64–77. 10.1016/j.ydbio.2016.07.023 27521050

[B26] Haider M. S. Zhang C. Kurjogi M. M. Pervaiz T. Zheng T. Zhang C. (2017). Insights into grapevine defense response against drought as revealed by biochemical, physiological and RNA-Seq analysis. Sci. Rep. 7, 1–15. 10.1038/s41598-017-13464-3 29030640 PMC5640638

[B27] Haider M. S. Kurjogi M. M. Khalil-ur-Rehman M. Pervez T. Songtao J. Fiaz M. (2018). Drought stress revealed physiological, biochemical and gene-expressional variations in ‘Yoshihime' peach (*Prunus persica* L) cultivar. J. Plant Interact. 13, 83–90. 10.1080/17429145.2018.1432772

[B28] Hou S. Zhu G. Li Y. Li W. Fu J. Niu E. (2018). Genome-wide association studies reveal genetic variation and candidate genes of drought stress related traits in cotton (*Gossypium hirsutum* L.). Front. Plant Sci. 9, 1–15. 10.3389/fpls.2018.01276 30233620 PMC6129771

[B29] Huang D. Wu W. Abrams S. R. Cutler A. J. (2008). The relationship of drought-related gene expression in *Arabidopsis thaliana* to hormonal and environmental factors. J. Exp. Bot. 59, 2991–3007. 10.1093/jxb/ern155 18552355 PMC2504347

[B30] Hwang J. Lim C. Chen H. Je J. Song C. Lim C. (2012). Overexpression of Arabidopsis dehydration-responsive element-binding protein 2C confers tolerance to oxidative stress. Mol. Cells 33, 135–140. 10.1007/s10059-012-2188-2 22286229 PMC3887724

[B31] Iacono F. Buccella A. Peterlunger E. (1998). Water stress and rootstock influence on leaf gas exchange of grafted and ungrafted grapevines. Sci. Hortic. (Amsterdam). 75, 27–39. 10.1016/S0304-4238(98)00113-7

[B32] IPCC (2014). “Climate Change 2014 Synthesis Report,” in Contribution of Working Groups I, II and III to the Fifth Assessment Report of the Intergovernmental Panel on Climate Change. Eds. Pachauri R. K. Meyer Geneva L. A. (Switzerland: Intergovernmental Panel on Climate Change (IPCC)).

[B33] IPCC (2018). “Global warming of 1.5°C,” in An IPCC Special Report on the impacts of global warming of 1.5°C above pre-industrial levels and related global greenhouse gas emission pathways, in the context of strengthening the global response to the threat of climate change, sustainable development. Intergovernmental Panel on Climate Change (IPCC). Masson-Delmotte T. W. V. Zhai P. PörtnerV. Masson-Delmotte H. O. Zhai P. Pörtner H. O. Roberts D. Skea J. Shukla P. R. Pirani A. Moufouma-Okia W. Péan C. Pidcock R. Connors S. Matthews J. B. R. Chen Y. Zhou X. Gomis M. I. Lonnoy E. Maycock T.

[B34] Irigoyen J. J. Goicoechea N. Antolín M. C. Pascual I. Sánchez-Díaz M. Aguirreolea J. (2014). Growth, photosynthetic acclimation and yield quality in legumes under climate change simulations : an updated survey. Plant Sci. 226, 22–29. 10.1016/j.plantsci.2014.05.008 25113447

[B35] Jiménez S. Dridi J. Gutiérrez D. Moret D. Irigoyen J. J. Moreno M. A. (2013). Physiological, biochemical and molecular responses in four *Prunus* rootstocks submitted to drought stress. Tree Physiol. 33, 1061–1075. 10.1093/treephys/tpt074 24162335

[B36] Jones H. G. (2007). Monitoring plant and soil water status: Established and novel methods revisited and their relevance to studies of drought tolerance. J. Exp. Bot. 58, 119–130. 10.1093/jxb/erl118 16980592

[B37] Kelly J. W. G. Duursma R. A. Atwell B. J. Tissue D. T. Medlyn B. E. (2016). Drought × CO_2_ interactions in trees: a test of the low-intercellular CO_2_ concentration (Ci) mechanism. New Phytol. 209, 1600–1612. 10.1111/nph.13715 26526873

[B38] Kizildeniz T. Mekni I. Santesteban H. Pascual I. Morales F. Irigoyen J. J. (2015). Effects of climate change including elevated CO_2_ concentration, temperature and water deficit on growth, water status, and yield quality of grapevine (*Vitis vinifera* L.) cultivars. Agric. Water Manage. 159, 155–164. 10.1016/j.agwat.2015.06.015

[B39] Kizildeniz T. Pascual I. Irigoyen J. J. Morales F. (2018). Using fruit-bearing cuttings of grapevine and temperature gradient greenhouses to evaluate effects of climate change (elevated CO_2_ and temperature, and water deficit) on the cv. red and white Tempranillo. Yield and must quality in three consecutive growin. Agric. Water Manage. 202, 299–310. 10.1016/j.agwat.2017.12.001

[B40] Krasensky J. Jonak C. (2012). Drought, salt, and temperature stress-induced metabolic rearrangements and regulatory networks. J. Exp. Bot. 63, 1593–1608. 10.1093/jxb/err460 22291134 PMC4359903

[B41] Ksouri N. Jiménez S. Wells C. E. Contreras-Moreira B. Gogorcena Y. (2016). Transcriptional responses in root and leaf of *Prunus persica* under drought stress using RNA sequencing. Front. Plant Sci. 7, 1715. 10.3389/fpls.2016.01715 27933070 PMC5120087

[B42] Leakey A. D. B. Ainsworth E. A. Bernacchi C. J. Rogers A. Long S. P. Ort D. R. (2009). Elevated CO_2_ effects on plant carbon, nitrogen, and water relations: Six important lessons from FACE. J. Exp. Bot. 60, 2859–2876. 10.1093/jxb/erp096 19401412

[B43] Leibar U. Aizpurua A. Unamunzaga O. Pascual I. Morales F. (2015). How will climate change influence grapevine cv. Tempranillo photosynthesis under different soil textures? Photosynth. Res. 124, 199–215. 10.1007/s11120-015-0120-2 25786733

[B44] Liu T. Longhurst A. D. Talavera-Rauh F. Hokin S. A. Barton M. K. (2016). The Arabidopsis transcription factor ABIG1 relays ABA signaled growth inhibition and drought induced senescence. Elife 5, 1–19. 10.7554/eLife.13768 PMC5050019 27697148

[B45] Lo Bianco R. Rieger M. Sung S. J. S. (2000). Effect of drought on sorbitol and sucrose metabolism in sinks and sources of peach. Physiol. Plant 108, 71–78. 10.1034/j.1399-3054.2000.108001071.x

[B46] Long S. P. Ainsworth E. A. Rogers A. Ort D. R. (2004). Rising atmospheric carbon dioxide: Plants FACE the future. Annu. Rev. Plant Biol. 55, 591–628. 10.1146/annurev.arplant.55.031903.141610 15377233

[B47] Long S. P. Ainsworth E. A. Leakey A. D. B. Nösberger J. Ort D. R. (2006). Food for thought: lower-than-expected crop yield stimulation with rising CO_2_ concentrations. Science (80-.), 1918–1921. 10.1126/science.1114722 16809532

[B48] Lovisolo C. Perrone I. Carra A. Ferrandino A. Flexas J. Medrano H. (2010). Drought-induced changes in development and function of grapevine (*Vitis* spp.) organs and in their hydraulic and non-hydraulic interactions at the whole-plant level: a physiological and molecular update. Funct. Plant Biol. 37, 98. 10.1071/FP09191

[B49] Ma Q. J. Sun M. H. Lu J. Liu Y. J. Hu D. G. Hao Y. J. (2017). Transcription factor AREB2 is involved in soluble sugar accumulation by activating sugar transporter and amylase genes. Plant Physiol. 174, 2348–2362. 10.1104/pp.17.00502 28600345 PMC5543958

[B50] Madhu M. Hatfeld J. L. (2013). Dynamics of plant root growth under increased atmospheric carbon dioxide. Agron. J. 105, 657–669. 10.2134/agronj2013.0018

[B51] Martínez-Lüscher J. Kizildeniz T. Vučetić V. Dai Z. Luedeling E. van Leeuwen C. (2016). Sensitivity of grapevine phenology to water availability, temperature and CO_2_ concentration. Front. Environ. Sci. 4, 48. 10.3389/fenvs.2016.00048

[B52] Medina S. Vicente R. Amador A. Araus J. L. (2016). Interactive effects of elevated [CO_2_] and water stress on physiological traits and gene expression during vegetative growth in four durum wheat genotypes. Front. Plant Sci. 7, 1–17. 10.3389/fpls.2016.01738 27920787 PMC5118623

[B53] Meggio F. Prinsi B. Negri A. Simone di Lorenzo G. Lucchini G. Pitacco A. (2014). Biochemical and physiological responses of two grapevine rootstock genotypes to drought and salt treatments. Aust. J. Grape Wine Res. 20, 310–323. 10.1111/ajgw.12071

[B54] Meisel L. Fonseca B. Gonzalez S. Baeza-Yates R. Cambiazo V. Campos R. (2005). A rapid and efficient method for purifying high quality total RNA from peaches (*Prunus persica*) for functional genomics analyses. Biol. Res. 38, 83–88. 10.4067/S0716-97602005000100010 15977413

[B55] Mellisho C. D. Cruz Z. N. Conejero W. Ortuno M. F. Rodríguez P. (2011). Mechanisms for drought resistance in early maturing cvar Flordastar peach trees. J. Agric. Sci. 149, 609–616. 10.1017/S0021859611000141

[B56] Miller G. Honig A. Stein H. Suzuki N. Mittler R. Zilberstein A. (2009). Unraveling delta1-pyrroline-5- carboxylate-proline cycle in plants by uncoupled expression of proline oxidation enzymes. J. Biol. Chem. 284, 26482–26492. 10.1074/jbc.M109.009340 19635803 PMC2785336

[B57] Moing A. Maucourt M. Renaud C. Gaudillère M. Brouquisse R. Lebouteiller B. (2004). Quantitative metabolic profiling by 1-dimensional 1H-NMR analyses: application to plant genetics and functional genomics. Funct. Plant Biol. 31, 889–902. 10.1071/FP04066 32688957

[B58] Morales F. Pascual I. Sánchez-Díaz M. Aguirreolea J. Irigoyen J. J. Goicoechea N. (2014). Methodological advances: Using greenhouses to simulate climate change scenarios. Plant Sci. 226, 30–40. 10.1016/j.plantsci.2014.03.018 25113448

[B59] Morgan J. (1984). Osmoregulation and water stress in higher plants. Annu. Rev. Plant Physiol. 35, 299–319. 10.1146/annurev.pp.35.060184.001503

[B60] Nakashima K. Yamaguchi-Shinozaki K. Shinozaki K. (2014). The transcriptional regulatory network in the drought response and its crosstalk in abiotic stress responses including drought, cold, and heat. Front. Plant Sci. 5, 1–7. 10.3389/fpls.2014.00170 PMC4032904 24904597

[B61] Nakhforoosh A. Grausgruber H. Kaul H. P. Bodner G. (2015). Dissection of drought response of modern and underutilized wheat varieties according to passioura's yield-water framework. Front. Plant Sci. 6, 1–13. 10.3389/fpls.2015.00570 26257766 PMC4511830

[B62] NOAA Mauna Loa Atmospheric Baseline Observatory (2019). Carbon dioxide levels hit record peak in May. Available at: https://research.noaa.gov/article/ArtMID/587/ArticleID/2461/Carbon-dioxide-levels-hit-record-peak-in-May.

[B63] Oh S.-J. Song S. I. Kim Y. S. Jang H.-J. Kim S. Y. Kim M. (2005). Arabidopsis CBF3/DREB1A and ABF3 in transgenic rice increased tolerance to abiotic stress without stunting growth. Plant Physiol. 138, 341–351. 10.1104/pp.104.059147 15834008 PMC1104188

[B64] Pérez-Jiménez M. Hernández-Munuera M. Piñero M. C. López-Ortega G. del Amor F. M. (2018). Are commercial sweet cherry rootstocks adapted to climate change? Short-term waterlogging and CO_2_ effects on sweet cherry cv. ‘Burlat.’. Plant Cell Environ. 41, 908–918. 10.1111/pce.12920 28107563

[B65] Pazzagli P. T. Weiner J. Liu F. (2016). Effects of CO_2_ elevation and irrigation regimes on leaf gas exchange, plant water relations, and water use efficiency of two tomato cultivars. Agric. Water Manage. 169, 26–33. 10.1016/j.agwat.2016.02.015

[B66] Pedroso F. K. J. V. Prudente D. A. Bueno A. C. R. Machado E. C. Ribeiro R. V. (2014). Drought tolerance in citrus trees is enhanced by rootstock-dependent changes in root growth and carbohydrate availability. Environ. Exp. Bot. 101, 26–35. 10.1016/j.envexpbot.2013.12.024

[B67] Ranney T. G. Bassuk N. L. Whitlow T. H. (1991). Osmotic adjustment and solute constituents in leaves and roots of water-stressed cherry (*Prunus*) trees. J. Am. Soc Hortic. Sci. 116, 684–688. 10.21273/JASHS.116.4.684

[B68] Ruijter J. M. Ramakers C. Hoogaars W. M. H. Karlen Y. Bakker O. Van den Hoff M. J. B. (2009). Amplification efficiency: linking baseline and bias in the analysis of quantitative PCR data. Nucleic Acids Res. 37, e45. 10.1093/nar/gkp045 19237396 PMC2665230

[B69] Sakanishi K. Kanayama Y. Mori H. Yamada K. Yamaki S. (1998). Expression of the gene for NADH-dependent sorbitol-6-phosphate dehy-drogenase in peach leave of various developmental stages. Plant Cell Physiol. 39, 1372–1374. 10.1093/oxfordjournals.pcp.a029344

[B70] Salazar-Parra C. Aguirreolea J. Sánchez-Díaz M. Irigoyen J. J. Morales F. (2012). Climate change (elevated CO_2_, elevated temperature and moderate drought) triggers the antioxidant enzymes' response of grapevine cv. Tempranillo, avoiding oxidative damage. Physiol. Plant 144, 99–110. 10.1111/j.1399-3054.2011.01524.x 21929631

[B71] Salazar-Parra C. Aranjuelo I. Pascual I. Erice G. Sanz-Sáez Á. Aguirreolea J. (2015). Carbon balance, partitioning and photosynthetic acclimation in fruit-bearing grapevine (*Vitis vinifera* L. cv. Tempranillo) grown under simulated climate change (elevated CO_2_, elevated temperature and moderate drought) scenarios in temperature gradient greenhouse. J. Plant Physiol. 174, 97–109. 10.1016/j.jplph.2014.10.009 25462972

[B72] Salazar-Parra C. Aranjuelo I. Pascual I. Aguirreolea J. Sánchez-Díaz M. Irigoyen J. J. (2018). Is vegetative area, photosynthesis, or grape C uploading involved in the climate change-related grape sugar/anthocyanin decoupling in Tempranillo? Photosynth. Res. 138, 115–128. 10.1007/s11120-018-0552-6 29980966

[B73] Sami F. Yusuf M. Faizan M. Faraz A. Hayat S. (2016). Role of sugars under abiotic stress. Plant Physiol. Biochem. 109, 54–61. 10.1016/j.plaphy.2016.09.005 27639065

[B74] Santana-Veira D. D. S. Freschi L. Aragão L. Henrique D. Moraes S. De Neves D. M. (2016). Survival strategies of citrus rootstocks subjected to drought. Sci. Rep. 6, 1–12. 10.1038/srep38775 27996018 PMC5171762

[B75] Sanz-Sáez Á. Erice G. Aguirreolea J. Irigoyen J. J. Sánchez-Díaz M. (2012). Alfalfa yield under elevated CO_2_ and temperature depends on the *Sinorhizobium* strain and growth season. Environ. Exp. Bot. 77, 267–273. 10.1016/j.envexpbot.2011.11.017

[B76] Sanz-Sáez Á. Erice G. Aranjuelo I. Aroca R. Ruíz-Lozano J. M. Aguirreolea J. (2013). Photosynthetic and molecular markers of CO_2_-mediated photosynthetic downregulation in nodulated alfalfa. J. Integr. Plant Biol. 55, 721–734. 10.1111/jipb.12047 23480453

[B77] Scarascia-Mugnozza G. Calfapietra C. Ceulemans R. Gielen B. Cotrufo M. De Angelis P. (2006). “Responses to elevated [CO_2_] of a short rotation, multispecies poplar plantation: the POPFACE/EUROFACE experiment,” in Managed ecosystems and CO_2_: case studies, processes and perspectives. Ecological Studies 187. Eds. Long S. Norby R. Stitt M. Hendrey G. Blum H. (Berlin, Heidelberg, Germany: Springer-Verlag), 173–195. 10.1007/3-540-31237-4_10

[B78] Schefe J. H. Lehmann K. E. Buschmann I. R. Unger T. Funke-Kaiser H. (2006). Quantitative real-time RT-PCR data analysis: Current concepts and the novel “gene expression's CT difference” formula. J. Mol. Med. 84, 901–910. 10.1007/s00109-006-0097-6 16972087

[B79] Serra I. Strever A. Myburgh P. A. Deloire A. (2014). Review: The interaction between rootstocks and cultivars (*Vitis vinifera* L.) to enhance drought tolerance in grapevine. Aust. J. Grape Wine Res. 20, 1–14. 10.1111/ajgw.12054

[B80] Solari L. I. Johnson S. Dejong T. M. (2006). Hydraulic conductance characteristics of peach (*Prunus persica*) trees on different rootstocks are related to biomass production and distribution. Tree Physiol. 26, 1343–1350. 10.1093/treephys/26.10.1343 16815836

[B81] Song Y. Chen Q. Ci D. Shao X. Zhang D. (2014). Effects of high temperature on photosynthesis and related gene expression in poplar. BMC Plant Biol. 14, 111. 10.1186/1471-2229-14-111 24774695 PMC4036403

[B82] Suzuki Y. Dandekar A. M. (2014). Sucrose induces expression of the sorbitol-6-phosphate dehydrogenase gene in source leaves of loquat. Physiol. Plant 150, 355–362. 10.1111/ppl.12106 24102486

[B83] Szabados L. Savoure A. (2010). Proline: a multifunctional amino acid. Trends Plant Sci. 15, 89–97. 10.1016/j.tplants.2009.11.009 20036181

[B84] Turner N. (1986). Adaptation to water deficits: a changing perspective. Aust. J. Plant Physiol. 13, 175–190. 10.1071/PP9860175

[B85] Untergasser A. Nijveen H. Rao X. Bisseling T. Geurts R. Leunissen J. A. M. (2007). Primer3Plus, an enhanced web interface to Primer3. Nucleic Acids Res. 35, 71–74. 10.1093/nar/gkm306 PMC1933133 17485472

[B86] Verbruggen N. Hermans C. (2008). Proline accumulation in plants: a review. Amino Acids 35, 753–759. 10.1007/s00726-008-0061-6 18379856

[B87] Vicente R. Pérez P. Martínez-Carrasco R. Usadel B. Kostadinova S. Morcuende R. (2015). Quantitative RT-PCR platform to measure transcript levels of C and N metabolism-related genes in durum wheat: transcript profiles in elevated [CO_2_] and high temperature at different nitrogen supplies. Plant Cell Physiol. 56, 1556–1573. 10.1093/pcp/pcv079 26063390

[B88] Wu B. H. Li S. H. Nosarzewski M. Archbold D. D. (2010). Sorbitol dehydrogenase gene expression and enzyme activity in apple: Tissue specificity during bud development and response to rootstock vigor and growth manipulation. J. Am. Soc Hortic. Sci. 135, 379–387. 10.21273/JASHS.135.4.379

[B89] Yu J. Sun L. Fan N. Yang Z. Huang B. (2015). Physiological factors involved in positive effects of elevated carbon dioxide concentration on Bermuda grass tolerance to salinity stress. Environ. Exp. Bot. 115, 20–27. 10.1016/j.envexpbot.2015.02.003

