# Supplementary material for: Interactional Effects of Climate Change Factors on the Water Status, Photosynthetic Rate, and Metabolic Regulation in Peach
Source: Front Plant Sci. 2020 Feb 28;11:43. doi: 10.3389/fpls.2020.00043 (PMC7059187; doi:10.3389/fpls.2020.00043)
Supplement: Supplementary file 1 [file Table_1.pdf]

Table S1. List of primer sequences and amplicon size of differentially expressed genes in roots (stocks GF677 and Adesoto) and leaves (graft, cv. Catherina) selected for RT-qPCR. Abbreviation: GDR Genome Database of Rosaceae.

Gene abbreviation	Gene Description	ID in GDR or NBI Database	Primer sequences Seq (5'->3')	Amplicon size (bp)
SDH	Sorbitol dehydrogenase	ppa007458m_F	CGAAGTTGGTAGCTTGGTGAAGA	91
		ppa007458m_R	CTTGCACTGCTCACATCTCCA	
S6PDH	Sorbitol-6-phosphate dehydrogenase	ppa009007m_F	ACATGGCACGACATGGAAAAGAC	128
		ppa009007m_R	AATTGGCTCACTTGAGGCTTGAT	
SIP1	Raffinose synthase	ppa001744m_F	GGTGCCATCCAGTCCTTTGT	121
		ppa001744m_R	TGCCCTCAATCCTGCAACTT	
P5CS	Δ-1-pyrrolyne-carboxylate synthase	GeneBank Number	CCAAGGGGCAGCAATAAACTG	439
		XP008233492.	CTTCTAGGTCTTCTGCGATAA	
P5CR	Δ-1-pyrrolyne-carboxylate reductase	GeneBank Number	GCATCCAGGTCAGCTAAAGG	206
		XP008240728.	CCAGCGCTATGAAAAGGAAG	
OAT	Ornithine aminotransferase	GeneBank Number	GTCAGAGAGCTGTGCTCAAA	389
		XM008227284.1	ACCTCTTCCTCGAACTTCCT	
PIP2	Phosphatidylinositol 4,5-bisphosphate	ppa009630m_F	TACTGCACTGCTGGCATCTCC	165
	plasma intrinsic protein	ppa009630m_R	ACATATGGCTCCCAAGGACTGA	
DREB2	Dehydration responsive element binding protein 2	ppa007606m_F	CAAGCCCTCGTGAAGGAAGA	167
		ppa007606m_R	ACATCAAACACCTCCTCCTCTGT	
AREB2	ABA responsive element binding protein 2	ppa007729m_F	CACAACAGCAGCCACTCTTCC	105
		ppa007729m_R	TTGGTCCCCTGGTTCTTGG	
HAT22	Homeodomain-leucine zipper protein	ppa009614m_F	GTCTCACAACATCGTCCCCTCAAG	145
		ppa009614m_R	GGCTCACCAGGAAGACCCAAAG	
Actin 2	House keeping	ppa007238m_F	ACTGGACGACATGGAAAAG	102
		ppa007238m_R	GATTCAGGGGTGCCTCAGTA	
