# Supplementary material for: Interactional Effects of Climate Change Factors on the Water Status, Photosynthetic Rate, and Metabolic Regulation in Peach
Source: Front Plant Sci. 2020 Feb 28;11:43. doi: 10.3389/fpls.2020.00043 (PMC7059187; doi:10.3389/fpls.2020.00043)
Supplement: Supplementary file 2 [file Table_2.pdf]

Supplementary Table 2. Root soluble sugars and proline (mg g^{-1} DW) concentration (n=4) in ambient (amb CO_2) and high (CO_2 elev) CO_2, ambient (T^e amb) and high (T^e amb + 4ºC) temperature, and control irrigation and drought stressed GF677 *Prunus* rootstock budded with cv. Catherina, after 23 days of treatment.

Roots GF 677			Fructose	Glucose	Raffinose	Sucrose	Sorbitol	Xylose	Total sugars	Proline
**Principal Effects**										
CO_2		CO_2 Amb.	4.5	11.9 **b**	1.3	17.6	14.2	0.6 **b**	50.1 **b**	1.0
		CO_2 Elev.	5.4	20.0 **a**	1.2	21.0	13.7	1.0 **a**	62.2 **a**	1.3
T^e		T^e Amb.	4.4	14.6	1.2	20.5	13.6	0.9	55.1	1.0
		T^e Amb+4ºC	5.6	17.5	1.3	18.2	14.2	0.8	57.5	1.4
Irrigation		Control	4.7	14.6	0.9 **b**	18.3	16.2 **a**	0.8	55.5	0.7 **b**
		Drought	5.3	17.4	1.6 **a**	20.3	11.7 **b**	0.8	57.1	1.7 **a**
Interaction										
CO_2 Amb		T^e Amb	4.2	11.5	1.3	18.4	13.1	1.1	49.2	1.0
		T^e Amb+4ºC	4.8	12.2	1.3	16.9	15.1	1.2	50.9	1.2
CO_2 Elev		T^e Amb	4.6	17.3	1.0	22.4	14.1	1.3	60.3	1.1
		T^e Amb+4ºC	6.4	22.6	1.3	19.5	13.2	1.3	64.1	1.7
CO_2 Amb		Control	4.5	11.8	0.7	16.2	16.3	1.0	50.2	1.3
		Drought	4.9	17.1	1.1	20.1	16.2	1.2	60.2	1.9
CO_2 Elev		Control	4.5	11.9	1.8	18.8	12.3	1.3	50	0.9
		Drought	6.0	22.9	1.3	21.8	11.1	1.4	64.2	0.9
T^e Amb		Control	4.0	12.7	1.1 **b**	20.0	16.7	1.0 **a**	55.5	0.9
		Drought	5.3	16.3	0.7 **b**	16.8	15.9	0.6 **b**	55.6	0.9
T^e Amb+4ºC		Control	4.7	16.2	1.2 **b**	20.9	10.9	0.7 **b**	54.8	1.2
		Drought	5.8	18.6	1.9 **a**	19.7	12.5	0.8 **ab**	59.4	2.0
CO_2 Amb	T^e Amb.	Control	3.7	10.9	1.1	17.0	16.3	1.0	49.8	0.6
		Drought	4.6	11.9	1.5	19.6	10.7	0.6	48.7	1.1
	T^e Amb+4ºC	Control	5.1	12.5	0.5	15.7	16.3	0.3	50.5	0.7
		Drought	4.5	12.0	2.1	18.1	14.0	0.7	51.3	1.5
CO_2 Elev.	T^e Amb.	Control	4.2	14.1	1.1	22.3	17.0	1.1	59.8	0.7
		Drought	4.9	20.5	1.0	22.4	11.2	0.9	60.9	1.3
	T^e Amb+4ºC	Control	5.5	20.0	0.9	17.8	15.5	1.0	60.7	0.6
		Drought	7.2	25.3	1.7	21.3	11.0	1.1	67.5	2.6
Signification										
CO_2			ns	***	ns	ns	ns	**	**	ns
T^e			ns	ns	ns	ns	ns	ns	ns	ns
Irrigation			ns	ns	**	ns	***	ns	ns	***
$CO_2 \times T^e$			ns	ns	ns	ns	ns	ns	ns	ns
CO_2 × Irrigation			ns	ns	ns	ns	ns	ns	ns	ns
T^e × Irrigation			ns	ns	**	ns	ns	*	ns	ns
$CO_2 \times T^e$ × Irrigation			ns	ns	ns	ns	ns	ns	ns	ns

Three-way ANOVA was performed for linear model, on raw data. Significance: $*P \le 0.05$, $**P \le 0.01$, $***P \le 0.001$ and ns indicates not significant. Comparison means by Duncan's test ($P \le 0.05$) were shown for the significant interaction among treatments. Different letters indicate significant differences among data within the same factor or interaction. Amb= Ambient, Elev= Elevated; T^e= Temperature.
