# Supplementary material for: Interactional Effects of Climate Change Factors on the Water Status, Photosynthetic Rate, and Metabolic Regulation in Peach
Source: Front Plant Sci. 2020 Feb 28;11:43. doi: 10.3389/fpls.2020.00043 (PMC7059187; doi:10.3389/fpls.2020.00043)
Supplement: Supplementary file 3 [file Table_3.pdf]

Supplementary Table 3. Root soluble sugars and proline (mg g^{-1} DW) concentration (n=4) in ambient (amb CO_2) and high (CO_2 elev) CO_2, ambient (T^e amb) and high (T^e amb + 4ºC) temperature, and control irrigation and drought stressed Adesoto *Prunus* rootstock budded with cv. Catherina, after 23 days of treatment.

Roots Adesoto			Fructose	Glucose	Raffinose	Sucrose	Sorbitol	Xylose	Total sugars	Proline
**Principal Effects**										
CO_2		CO_2 Amb.	4.7 **b**	18.7 **b**	0.6	26.3	18.6	0.9	70.5 **b**	0.9
		CO_2 Elev.	5.8 **a**	23.6 **a**	0.7	30.5	20.3	1.1	82.7 **a**	1.0
T^e		T^e Amb.	5.0	21.4	0.7	27.4	15.3 **b**	0.9	71.3 **b**	0.9
		T^e Amb+4ºC	5.4	21.0	0.7	29.5	23.3 **a**	1.1	81.4 **a**	1.0
Irrigation		Control	5.9 **a**	19.7	0.6	30.7	14.2 **b**	1.1	72.6	0.9
		Drought	4.6 **b**	22.6	0.8	26.4	24.4 **a**	1.0	80.2	1.0
Interaction										
CO_2 Amb		T^e Amb	4.8	19.2	0.7	24.8	14.4	0.9	64.8	0.6
		T^e Amb+4ºC	4.6	18.1	0.6	27.7	22.3	0.9	74.1	0.7
CO_2 Elev		T^e Amb	5.3	23.3	0.6	29.7	16.1	1.0	76.1	0.7
		T^e Amb+4ºC	6.2	23.8	0.8	31.4	24.4	1.3	87.9	0.8
CO_2 Amb		Control	5.0	20.2 **b**	0.6	26.3	13.4	1.0	66.5	0.7
		Drought	6.6	19.2 **b**	0.6	34.5	14.9	1.2	77	0.9
CO_2 Elev		Control	4.4	17.3 **b**	0.7	26.3	23.2	0.8	72.7	0.6
		Drought	4.9	27.9 **a**	0.8	26.5	25.6	1.1	86.9	0.7
T^e Amb		Control	5.5	19.1	0.6	29.5	9.6	1.1	65.1	0.8
		Drought	4.7	23.5	0.7	25.6	20.3	0.9	75.8	0.6
T^e Amb+4ºC		Control	6.2	20.2	0.6	31.8	18.3	1.2	78.3	0.8
		Drought	4.6	21.7	0.8	27.3	28.4	1.0	83.8	1.0
CO_2 Amb	T^e Amb.	Control	4.5	17.7	0.5	23.9	6.8	0.8	54.5	0.8
		Drought	5.0	20.4	0.8	25.5	20.1	1.0	73.3	0.7
	T^e Amb+4ºC	Control	5.4	22.0	0.7	28.1	18.3	1.2	76.8	0.7
		Drought	3.8	14.3	0.5	27.2	26.3	0.7	73.6	1.2
CO_2 Elev.	T^e Amb.	Control	6.2	20.1	0.6	33.7	11.6	1.1	73.1	1.0
		Drought	4.5	26.6	0.7	25.7	20.7	0.9	79.5	1.1
	T^e Amb+4ºC	Control	7.1	18.4	0.5	35.4	18.3	1.2	81.3	1.0
		Drought	5.4	29.2	1.0	27.4	30.5	1.4	95.4	1.1
Signification										
CO_2			**	*	ns	ns	ns	ns	*	ns
T^e			ns	ns	ns	ns	***	ns	*	ns
Irrigation			**	ns	ns	ns	***	ns	ns	ns
$CO_2 \times T^e$			ns	ns	ns	ns	ns	ns	ns	ns
CO_2 × Irrigation			ns	**	ns	ns	ns	ns	ns	ns
T^e × Irrigation			ns	ns	ns	ns	ns	ns	ns	ns
$CO_2 \times T^e$ × Irrigation			ns	ns	ns	ns	ns	ns	ns	ns

Three-way ANOVA was performed for linear model, on raw data. Significance: *$P \leq 0.05$, **$P \leq 0.01$, ***$P \leq 0.001$ and ns indicates not significant. Comparison means by Duncan's test ($P \leq 0.05$) were shown for the significant interaction among treatments. Different letters indicate significant differences among data within the same factor or interaction. Amb= Ambient, Elev= Elevated; T^e= Temperature.
