# Supplementary material for: Interactional Effects of Climate Change Factors on the Water Status, Photosynthetic Rate, and Metabolic Regulation in Peach
Source: Front Plant Sci. 2020 Feb 28;11:43. doi: 10.3389/fpls.2020.00043 (PMC7059187; doi:10.3389/fpls.2020.00043)
Supplement: Supplementary file 4 [file Table_4.pdf]

Supplementary Table 4. Scion leaf soluble sugars and proline (mg g^{-1} DW) concentration (n=4) in ambient (amb CO_2) and high (CO_2 elev) CO_2, ambient (T^e amb) and high (T^e amb + 4ºC) temperature, and control irrigation and drought stressed GF677 *Prunus* rootstock budded with cv. Catherina, after 23 days of treatment.

Leaves GF 677			Fructose	Glucose	Raffinose	Sucrose	Sorbitol	Xylose	Total sugars	Proline
**Principal Effects**										
CO_2		CO_2 Amb.	8.4 **b**	11.1 **b**	0.3 **b**	40.2 **b**	80.6 **b**	1.2	142.1 **b**	1.7 **b**
		CO_2 Elev.	10.3 **a**	14.2 **a**	0.4 **a**	60.5 **a**	90.9 **a**	1.3	177.3 **a**	3.1 **a**
T^e		T^e Amb.	9.7	11.4 **b**	0.4	54.6 **a**	83.0	1.2	160.2	2.9 **a**
		T^e Amb+4ºC	8.9	13.9 **a**	0.4	46.1 **b**	88.5	1.3	159.5	2.0 **b**
Irrigation		Control	10.2 **a**	12.1	0.4	53.4 **a**	79.4 **b**	1.1 **b**	157.7	2.2 **b**
		Drought	8.4 **b**	13.1	0.4	47.3 **b**	92.1 **a**	1.4 **a**	163.4	2.6 **a**
Interaction										
CO_2 Amb		T^e Amb	8.7	11.1	0.3	45.1	77.7	1.1	143.8	2.1
		T^e Amb+4ºC	8.2	11.2	0.3	35.3	83.5	1.2	139.7	1.4
CO_2 Elev		T^e Amb	10.8	11.8	0.4	64.2	88.3	1.3	176.7	3.7
		T^e Amb+4ºC	9.7	16.6	0.4	56.9	93.5	1.3	178.3	2.6
CO_2 Amb		Control	8.9	9.7	0.3	42.1	69.0 **b**	1.0	130.9	1.8 **bc**
		Drought	11.6	14.5	0.4	64.7	89.7 **a**	1.2	182.1	2.5 **b**
CO_2 Elev		Control	8.1	12.5	0.3	38.3	92.1 **a**	1.3	152.5	1.5 **c**
		Drought	8.9	13.8	0.4	56.3	92.0 **a**	1.4	172.8	3.6 **a**
T^e Amb		Control	10.6	10.0	0.4	57.6	80.7	1.0	160.3	2.7
		Drought	8.9	14.2	0.4	51.7	85.2	1.4	160.2	3.0
T^e Amb+4ºC		Control	9.9	12.7	0.4	49.2	78	1.2	152.8	1.8
		Drought	8.0	13.6	0.3	42.9	99.1	1.3	165.2	2.2
CO_2 Amb	T^e Amb.	Control	9.4	9.5	0.3	47.9	70.2	1.0	138.5	2.3
		Drought	8.0	12.5	0.3	42.4	85.2	1.3	149.8	1.9
	T^e Amb+4ºC	Control	8.3	9.9	0.3	36.3	67.8	1.0	124.9	1.6
		Drought	8.0	12.5	0.4	34.2	99.2	1.4	156.4	1.3
CO_2 Elev.	T^e Amb.	Control	11.9	10.6	0.4	67.4	91.2	1.1	182.2	3.1
		Drought	9.8	13.0	0.5	61.0	85.3	1.5	171.3	4.2
	T^e Amb+4ºC	Control	11.4	18.4	0.4	62.0	88.3	1.3	182.6	2.0
		Drought	8.0	14.7	0.4	51.7	98.8	1.3	175.3	3.1
Signification										
CO_2			**	**	**	***	*	ns	***	***
T^e			ns	*	ns	**	ns	ns	ns	***
Irrigation			**	ns	ns	*	*	**	ns	*
$CO_2 \times T^e$			ns	ns	ns	ns	ns	ns	ns	ns
CO_2 × Irrigation			ns	ns	ns	ns	*	ns	ns	**
T^e × Irrigation			ns	ns	ns	ns	ns	ns	ns	ns
$CO_2 \times T^e$ × Irrigation			ns	ns	ns	ns	ns	ns	ns	ns

Three-way ANOVA was performed for linear model, on raw data. Significance: *$P\leq0.05$, **$P\leq0.01$, ***$P\leq0.001$ and ns indicates not significant. Comparison means by Duncan's test ($P\leq0.05$) were shown for the significant interaction among treatments. Different letters indicate significant differences among data within the same factor or interaction. Amb= Ambient, Elev= Elevated; T^e= Temperature.
