# Supplementary material for: Interactional Effects of Climate Change Factors on the Water Status, Photosynthetic Rate, and Metabolic Regulation in Peach
Source: Front Plant Sci. 2020 Feb 28;11:43. doi: 10.3389/fpls.2020.00043 (PMC7059187; doi:10.3389/fpls.2020.00043)
Supplement: Supplementary file 5 [file Table_5.pdf]

Supplementary Table 5. Scion leaf soluble sugars and proline (mg g^{-1} DW) concentration (n=4) in ambient (amb CO_2) and high (CO_2 elev) CO_2, ambient (T^e amb) and high (T^e amb + 4ºC) temperature, and control irrigation and drought stressed Adesoto *Prunus* rootstock budded with cv. Catherina, after 23 days of treatment.

Leaves Adesoto			Fructose	Glucose	Raffinose	Sucrose	Sorbitol	Xylose	Total sugars	Proline
**Principal Effects**										
CO_2		CO_2 Amb.	9.1	15.9	1.0	39.0 **b**	91.1	1.4	157.2	2.6
		CO_2 Elev.	9.2	16.6	0.4	47.2 **a**	86.2	1.4	161.3	2.6
T^e		T^e Amb.	9.6	15.9	0.4	46.2	85.3	1.4	158.1	2.8
		T^e Amb+4ºC	8.8	16.6	0.9	40.5	91.0	1.4	159.4	2.4
Irrigation		Control	9.2	17.7	0.4	51.5 **a**	89.3	1.2 **b**	169.3	1.9 **b**
		Drought	9.2	15.0	1.0	35.5 **b**	87.4	1.6 **a**	149.1	3.2 **a**
Interaction										
CO_2 Amb		T^e Amb	9.8	19.2	0.7	41.7	86	1.4	155.2	2.6
		T^e Amb+4ºC	8.5	18.1	0.6	36.6	94.7	1.4	158.7	2.5
CO_2 Elev		T^e Amb	9.3	23.3	0.6	50	84	1.4	161.3	2.9
		T^e Amb+4ºC	9.2	23.8	0.8	44.4	87.2	1.4	159.7	2.2
CO_2 Amb		Control	9.5	20.2	0.6	48	90.1	1.2	159.1	1.8
		Drought	8.9	19.2	0.6	54.5	84.4	1.2	139.8	2.1
CO_2 Elev		Control	8.8	17.3	0.7	31.1	81.2	1.6	161.9	3.2
		Drought	9.6	27.9	0.8	39.9	97.7	1.5	176.7	3.1
T^e Amb		Control	9.3	19.1	0.6	53.3	79.7	1.2	161.5	2.4
		Drought	9.8	23.5	0.7	40.0	89.5	1.5	155.8	3.2
T^e Amb+4ºC		Control	9.1	20.2	0.6	49.9	96.9	1.2	175.3	1.6
		Drought	8.6	21.7	0.8	31.1	85.0	1.6	143.1	3.1
CO_2 Amb	T^e Amb.	Control	9.4	17.7	0.4	44.8 **ab**	76.1 **b**	1.2	150.1 **ab**	1.9
		Drought	10.2	14.3	0.5	39.5 **b**	93.2 **ab**	1.6	159.3 **ab**	3.1
	T^e Amb+4ºC	Control	9.6	21.6	0.4	50.5 **ab**	114.4 **a**	1.2	197.0 **a**	1.7
		Drought	7.4	10.5	2.6	22.7 **c**	75.3 **b**	1.5	120.4 **b**	3.4
CO_2 Elev.	T^e Amb.	Control	9.3	17.4	0.5	59.6 **ab**	82.1 **ab**	1.3	170.6 **ab**	2.7
		Drought	9.4	14.8	0.5	40.5 **b**	86.7 **ab**	1.5	152.3 **ab**	3.2
	T^e Amb+4ºC	Control	8.5	14.0	0.4	49.4 **ab**	80.2 **b**	1.1	153.8 **ab**	1.5
		Drought	9.8	20.4	0.4	39.4 **b**	94.5 **ab**	1.7	166.2 **ab**	2.9
Signification										
CO_2			ns	ns	ns	*	ns	ns	ns	ns
T^e			ns	ns	ns	ns	ns	ns	ns	ns
Irrigation			ns	ns	ns	***	ns	***	ns	***
$CO_2 \times T^e$			ns	ns	ns	ns	ns	ns	ns	ns
CO_2 × Irrigation			ns	ns	ns	ns	ns	ns	ns	ns
T^e × Irrigation			ns	ns	ns	ns	ns	ns	ns	ns
$CO_2 \times T^e$ × Irrigation			ns	ns	ns	*	*	ns	*	ns

Three-way ANOVA was performed for linear model, on raw data. Significance: *$P \leq 0.05$, ***$P \leq 0.001$ and ns indicates not significant. Comparison means by Duncan's test ($P \leq 0.05$) were shown for the significant interaction among treatments. Different letters indicate significant differences among data within the same factor or interaction. Amb= Ambient, Elev= Elevated; T^e= Temperature.
