# Supplementary material for: Interactional Effects of Climate Change Factors on the Water Status, Photosynthetic Rate, and Metabolic Regulation in Peach
Source: Front Plant Sci. 2020 Feb 28;11:43. doi: 10.3389/fpls.2020.00043 (PMC7059187; doi:10.3389/fpls.2020.00043)
Supplement: Supplementary file 6 [file Table_6.pdf]

frontiers

Supplementary Table 6. Gene expression (Rnorm values) in root tissue (n=4) under ambient (amb CO_2) and high (CO_2 elev) CO_2, ambient (T^e amb) and high (T^e amb + 4ºC) temperature, and control irrigation and drought stressed GF677 *Prunus* rootstock budded with cv. Catherina, after 23 days of treatment.

**Roots GF 677**			***SDH***	***S6PDH***	***SIP1***	***P5CS***	***P5CR***	***PIP2***	***DREB2***	***AREB2***	***HAT22***
**Principal Effects**											
CO_2		CO_2 Amb.	9.5	0.03	1.1 **a**	0.5	1.8	0.1	0.4	0.2	1.4
		CO_2 Elev.	9.1	0.01	0.9 **b**	0.7	2.1	0.1	0.3	0.2	1.7
T^e		T^e Amb.	13.3 **a**	0.03 **a**	1.2	0.7 **a**	2.2	0.1	0.5 **a**	0.2 **a**	2.1 **a**
		T^e Amb+4ºC	5.3 **b**	0.01 **b**	0.7	0.5 **b**	1.7	0.1	0.2 **b**	0.1 **b**	1.0 **b**
Irrigation		Control	14.5 **a**	0.01 **b**	0.6	0.3 **b**	1.6 b	0.1	0.4	0.2	1.7
		Drought	4.02 **b**	0.03 **a**	1.4	0.9 **a**	2.3 a	0.1	0.4	0.2	1.3
Interaction											
CO_2 Amb		T^e Amb	14.2	0.05 **a**	0.7	0.6	1.9	0.3	0.6	0.2	1.9
		T^e Amb+4ºC	4.4	0.01 **b**	0.4	0.5	2.1	0.2	0.3	0.1	0.8
CO_2 Elev		T^e Amb	12.4	0.02 **b**	0.9	0.9	1.1	0.4	0.5	0.3	2.2
		T^e Amb+4ºC	5.4	0.01 **b**	0.4	0.6	1.5	0.1	0.2	0.1	1.2
CO_2 Amb		Control	16.2	0.01	0.7 **a**	0.3	1.7	0.3	0.6	0.2 **a**	1.6
		Drought	14.8	0.01	0.6 **a**	0.4	2.2	0.2	0.3	0.1 **b**	2.1
CO_2 Elev		Control	3.9	0.04	0.7 **b**	0.8	1.9	0.3	0.3	0.1 **b**	1.2
		Drought	4.2	0.02	0.6 **a**	1.1	2.6	0.3	0.4	0.2 **a**	1.4
T^e Amb		Control	21.2 **a**	0.02 **b**	0.9	0.4	1.6	0.3	0.7	0.3	2.5
		Drought	5.4 **bc**	0.05 **a**	0.7	0.2	1.9	0.4	0.2	0.2	1
T^e Amb+4ºC		Control	7.8 **b**	0.01 **b**	0.5	1.0	1.6	0.1	0.5	0.1	1.7
		Drought	2.7 **c**	0.01 **b**	0.4	0.8	2	0.2	0.2	0.1	1
CO_2 Amb	T^e Amb.	Control	22.9	0.02	0.9	0.4	2.3	0.3	0.9	0.3	2.2
		Drought	5.4	0.08	1.5	0.8	2.2	0.3	0.4	0.2	1.7
	T^e Amb+4ºC	Control	7.2	0.01	0.5	0.2	1.1	0.2	0.3	0.1	0.9
		Drought	2.4	0.01	1.3	0.7	1.6	0.2	0.3	0.1	0.7
CO_2 Elev.	T^e Amb.	Control	19.5	0.01	0.5	0.5	1.8	0.3	0.3	0.2	2.7
		Drought	5.4	0.02	2.1	1.2	2.6	0.5	0.6	0.3	1.7
	T^e Amb+4ºC	Control	8.5	0.01	0.4	0.2	1.3	0.1	0.2	0.1	1.2
		Drought	3.0	0.02	0.8	1.0	2.9	0.2	0.2	0.2	1.2
Signification											
CO_2			ns	ns	*	ns	ns	ns	ns	ns	ns
T^e			***	**	ns	*	ns	ns	*	***	***
Irrigation			***	**	ns	***	*	ns	ns	ns	ns
$CO_2 \times T^e$			ns	*	ns	ns	ns	ns	ns	ns	ns
CO_2 × Irrigation			ns	ns	*	ns	ns	ns	ns	*	ns
T^e × Irrigation			***	*	ns	ns	ns	ns	ns	ns	ns
$CO_2 \times T^e$ × Irrigation			ns	ns	ns	ns	ns	ns	ns	ns	ns

Three-way ANOVA was performed for lineal model on raw data. Significance: *$P \leq 0.05$, **$P \leq 0.01$, ***$P \leq 0.001$ and ns indicates not significant. Comparison means by Duncan's test ($P \leq 0.05$) were shown for the significant interaction among treatments. Different letters indicate significant differences among data within the same factor or interaction. Amb= Ambient, Elev= Elevated; T^e= Temperature.
