# Supplementary material for: Interactional Effects of Climate Change Factors on the Water Status, Photosynthetic Rate, and Metabolic Regulation in Peach
Source: Front Plant Sci. 2020 Feb 28;11:43. doi: 10.3389/fpls.2020.00043 (PMC7059187; doi:10.3389/fpls.2020.00043)
Supplement: Supplementary file 7 [file Table_7.pdf]

Supplementary Table 7. Gene expression (Rnorm values) in root tissue (n=4) under ambient (amb CO_2) and high (CO_2 elev) CO_2, ambient (T^e amb) and high (T^e amb + 4ºC) temperature, and control irrigation and drought stressed Adesoto *Prunus* rootstock budded with cv. Catherina, after 23 days of treatment.

Roots Adesoto			*SDH*	*S6PDH*	*SIP1*	*P5CS*	*P5CR*	*PIP2*	*DREB2*	*AREB2*	*HAT22*
**Principal Effects**											
CO_2		CO_2 Amb.	7.8	0.004 **b**	0.5	0.4	1.2	0.11	0.6	0.14 **b**	1.3
		CO_2 Elev.	10.8	0.034 **a**	0.9	0.8	1.9	0.11	0.7	0.22 **a**	1.8
T^e		T^e Amb.	10.0	0.005	0.7	0.5	1.4	0.12	0.8	0.17	1.6
		T^e Amb+4ºC	8.6	0.032	0.7	0.7	1.8	0.10	0.5	0.19	1.5
Irrigation		Control	12.8 **a**	0.006	0.6	0.4	1.6	0.12	0.6	0.18	1.2
		Drought	5.8 **b**	0.032	0.9	0.7	1.5	0.10	0.6	0.17	1.9
Interaction											
CO_2 Amb		T^e Amb	8.2	0.003	0.4	0.3	0.9	0.13	0.7	0.13	1.3
		T^e Amb+4ºC	7.7	0.005	0.4	0.4	0.8	0.10	0.5	0.15	1.3
CO_2 Elev		T^e Amb	11.8	0.008	0.7	0.6	1.4	0.11	0.9	0.22	2.0
		T^e Amb+4ºC	9.5	0.059	0.8	1.0	1.6	0.11	0.4	0.23	1.8
CO_2 Amb		Control	10.6	0.005	0.4	1.0	0.8	0.15 **a**	0.6	0.14	1.1
		Drought	15.1	0.008	0.7	0.5	1.6	0.08 **b**	0.7	0.22	1.4
CO_2 Elev		Control	4.8	0.004	0.4	0.5	1.0	0.07 **b**	0.6	0.13	1.5
		Drought	6.9	0.052	0.7	0.3	1.4	0.14 **a**	0.7	0.22	2.3
T^e Amb		Control	14.7	0.007	0.6	0.3	1.2	0.13	0.9	0.18	1.4
		Drought	5.9	0.005	0.5	0.7	1.3	0.10	0.7	0.17	1.9
T^e Amb+4ºC		Control	11.1	0.006	0.5	0.4	1.2	0.10	0.4	0.19	1.2
		Drought	6.1	0.059	0.6	0.9	1.2	0.11	0.6	0.19	1.9
CO_2 Amb	T^e Amb.	Control	11.5	0.002 **b**	0.4	0.2	1.4	0.19	0.8	0.13	1.0
		Drought	5.1	0.004 **b**	0.8	0.5	0.9	0.07	0.5	0.13	1.5
	T^e Amb+4ºC	Control	10.0	0.007 **b**	0.6	0.3	1.2	0.12	0.3	0.15	1.2
		Drought	4.6	0.004 **b**	0.5	0.5	1.4	0.06	0.6	0.13	1.5
CO_2 Elev.	T^e Amb.	Control	17.1	0.010 **b**	1.0	0.4	1.5	0.09	1.0	0.22	1.6
		Drought	6.5	0.005 **b**	0.8	0.8	1.8	0.12	0.8	0.21	2.3
	T^e Amb+4ºC	Control	12.5	0.006 **b**	0.3	0.6	2.5	0.06	0.4	0.23	1.1
		Drought	7.3	0.113 **a**	1.4	1.2	1.9	0.15	0.5	0.23	2.2
Signification											
CO_2			ns	**	ns	ns	ns	ns	ns	*	ns
T^e			ns	ns	ns	ns	ns	ns	ns	ns	ns
Irrigation			**	ns	ns	ns	ns	ns	ns	ns	ns
$CO_2 \times T^e$			ns	ns	ns	ns	ns	ns	ns	ns	ns
CO_2 × Irrigation			ns	ns	ns	ns	ns	**	ns	ns	ns
T^e × Irrigation			ns	ns	ns	ns	ns	ns	ns	ns	ns
$CO_2 \times T^e$ × Irrigation			ns	*	ns	ns	ns	ns	ns	ns	ns

Three-way ANOVA was performed for lineal model on raw data. Significance: **P*≤0.05, ***P*≤0.01, ****P*≤0.001 and ns indicates not significant. Comparison means by Duncan's test (*P*≤0.05) were shown for the significant interaction among treatments. Different letters indicate significant differences among data within the same factor or interaction. Amb= Ambient, Elev= Elevated; T^e= Temperature.
