# Supplementary material for: Interactional Effects of Climate Change Factors on the Water Status, Photosynthetic Rate, and Metabolic Regulation in Peach
Source: Front Plant Sci. 2020 Feb 28;11:43. doi: 10.3389/fpls.2020.00043 (PMC7059187; doi:10.3389/fpls.2020.00043)
Supplement: Supplementary file 8 [file Table_8.pdf]

Supplementary Table 8. Gene expression (Rnorm values) in leaf tissue (n=4) under ambient (amb CO_2) and high (CO_2 elev) CO_2, ambient (T^e amb) and high (T^e amb + 4ºC) temperature, and control irrigation and drought stressed GF677 *Prunus* rootstock budded with cv. Catherina, after 23 days of treatment.

Leaves GF 677			*SDH*	*S6PDH*	*SIP1*	*P5CS*	*P5CR*	*OAT*	*HAT22*
**Principal Effects**									
CO_2		CO_2 Amb.	0.3	161.3	5.2	5.5	10.3	32.2	8.3 **a**
		CO_2 Elev.	0.1	178.2	3.0	4.7	5.8	24.3	5.0 **b**
T^e		T^e Amb.	0.3	219.0	3.9	5.5	6.8	25.5	6.9
		T^e Amb+4ºC	0.1	120.5	4.4	4.7	9.3	30.9	6.4
Irrigation		Control	0.2	106.7 **b**	2.4 **b**	4.6	10.3	24.6	5.2 **b**
		Drought	0.2	232.8 **a**	5.8 **a**	5.7	5.9	31.9	8.2 **a**
Interaction									
CO_2 Amb		T^e Amb	0.5	179.2	3.4 **ab**	4.9 **b**	5.1 **b**	22.4	7.9
		T^e Amb+4ºC	0.2	140.5	7.1 **a**	6.3 **a**	15.6 **a**	41.9	8.6
CO_2 Elev		T^e Amb	0.1	255.9	4.3 **ab**	6.2 **a**	8.5 **ab**	28.7	5.3
		T^e Amb+4ºC	0.2	100.6	1.8 **b**	3.1 **b**	3.1 **b**	20.1	4.7
CO_2 Amb		Control	0.26	136.0	3.0	5.3	16.6 **a**	32.1	5.2 **b**
		Drought	0.1	69.1	1.8	4.0	5.2 **b**	19.8	5.1 **b**
CO_2 Elev		Control	0.32	178.3	7.9	5.9	5.6 **b**	38.8	11.4 **a**
		Drought	0.11	287.4	4.3	5.4	6.4 **b**	28.8	4.9 **b**
T^e Amb		Control	0.2	136.2	2.6	4.3	6.0 **ab**	17.3	4.9
		Drought	0.4	293.4	5.2	6.7	8.1 **ab**	35.9	5.4
T^e Amb+4ºC		Control	0.2	68.9	2.2	4.9	14.5 **a**	30.9	8.1
		Drought	0.0	172.2	6.6	4.6	4.2 **b**	31	7.5
CO_2 Amb	T^e Amb.	Control	0.2	202.0	3.1 **b**	3.9	6.2	21.0	5.3
		Drought	0.6	162.1	3.0 **b**	5.6	4.1	23.7	11.8
	T^e Amb+4ºC	Control	0.3	86.6	2.2 **b**	6.4	24.4	37.7	5.1
		Drought	0.1	194.5	1.4 **b**	6.2	6.7	46.3	11.2
CO_2 Elev.	T^e Amb.	Control	0.1	87.0	3.7 **b**	4.7	5.9	15.4	4.5
		Drought	0.2	424.8	11.0 **a**	7.8	11.0	41.9	6.1
	T^e Amb+4ºC	Control	0.1	51.2	6.4 **ab**	3.3	4.6	24.2	5.6
		Drought	0.1	149.9	2.2 **b**	3.1	1.7	15.7	3.8
Signification									
CO_2			ns	ns	ns	ns	ns	ns	**
T^e			ns	ns	ns	ns	ns	ns	ns
Irrigation			ns	*	**	ns	ns	ns	*
$CO_2 \times T^e$			ns	ns	*	*	**	ns	ns
CO_2 × Irrigation			ns	ns	ns	ns	*	ns	*
T^e × Irrigation			ns	ns	ns	ns	*	ns	ns
$CO_2 \times T^e$ × Irrigation			ns	ns	*	ns	ns	ns	ns

Three-way ANOVA was performed for lineal model on raw data. Significance: *P≤0.05, **P≤0.01 and ns indicates not significant. Comparison means by Duncan's test (P≤0.05) were shown for the significant interaction among treatments. Different letters indicate significant differences among data within the same factor or interaction. Amb= Ambient, Elev= Elevated; T^e= Temperature.
