# Supplementary material for: Interactional Effects of Climate Change Factors on the Water Status, Photosynthetic Rate, and Metabolic Regulation in Peach
Source: Front Plant Sci. 2020 Feb 28;11:43. doi: 10.3389/fpls.2020.00043 (PMC7059187; doi:10.3389/fpls.2020.00043)
Supplement: Supplementary file 9 [file Table_9.pdf]

Supplementary Table 9. Gene expression (Rnorm values) in leaf tissue (n=4) under ambient (amb CO_2) and high (CO_2 elev) CO_2, ambient (T^e amb) and high (T^e amb + 4ºC) temperature, and control irrigation and drought stressed Adesoto *Prunus* rootstock budded with cv. Catherina, after 23 days of treatment.

Leaves Adesoto			*SDH*	*S6PDH*	*SIP1*	*P5CS*	*P5CR*	*OAT*	*HAT22*
**Principal Effects**									
CO_2		CO_2 Amb.	0.009 **b**	44.1	0.8	0.9	1.9 **a**	9.8	2.6 **a**
		CO_2 Elev.	0.036 **a**	34.3	0.5	0.8	1.2 **b**	7.7	1.6 **b**
T^e		T^e Amb.	0.009 **b**	46.9	0.7	0.8	1.5	8.1	1.9
		T^e Amb+4ºC	0.036 **a**	31.5	0.6	0.9	1.7	9.3	2.3
Irrigation		Control	0.027	31.7	0.5	0.9	2.2 **a**	9.0	1.9
		Drought	0.018	46.7	0.9	0.7	1.0 **b**	8.4	2.3
Interaction									
CO_2 Amb		T^e Amb	0.008 **b**	52.1	0.7	0.8 **b**	1.3	8.0	2.1
		T^e Amb+4ºC	0.010 **b**	36.1	0.9	1.1 **a**	1.2	11.1	3.1
CO_2 Elev		T^e Amb	0.009 **b**	41.7	0.8	0.9 **ab**	2.3	8.2	1.7
		T^e Amb+4ºC	0.063 **a**	24.6	0.3	0.7 **b**	1.7	7.1	1.5
CO_2 Amb		Control	0.006	41.1	0.6	1.0	2.8	9.9	2.3
		Drought	0.042	22.3	0.3	0.9	1.8	8.0	1.5
CO_2 Elev		Control	0.011	44.8	1.1	0.8	1.3	9.4	2.9
		Drought	0.022	46.6	0.7	0.7	0.7	7.3	1.7
T^e Amb		Control	0.013	36.9	0.4	0.8	1.6 **b**	7.3	1.5
		Drought	0.036	26.1	0.5	1.1	2.7 **a**	8.9	2.3
T^e Amb+4ºC		Control	0.005	55.4	1.0	0.8	1.3 **b**	7.8	2.3
		Drought	0.029	36	0.8	0.7	0.7 **b**	10.4	2.4
CO_2 Amb	T^e Amb.	Control	0.011	40.1	0.4	0.5	1.5 **b**	5.3 **b**	1.4
		Drought	0.006	64.0	1.1	0.8	1.9 **b**	10.6 **ab**	2.8
	T^e Amb+4ºC	Control	0.004	41.9	0.8	1.4	3.7 **a**	14.6 **a**	3.1
		Drought	0.015	30.3	1.1	0.8	0.8 **b**	8.5 **ab**	3.1
CO_2 Elev.	T^e Amb.	Control	0.014	34.4	0.4	1.0	1.7 **b**	8.8 **ab**	1.6
		Drought	0.004	48.9	1.1	0.8	0.7 **b**	7.6 **b**	1.9
	T^e Amb+4ºC	Control	0.080	10.3	0.3	0.8	1.8 **b**	7.2 **b**	1.4
		Drought	0.046	43.6	0.3	0.6	0.7 **b**	6.9 **b**	1.5
Signification									
CO_2			*	ns	ns	ns	*	ns	*
T^e			*	ns	ns	ns	ns	ns	ns
Irrigation			ns	ns	ns	ns	**	ns	ns
$CO_2 \times T^e$			*	ns	ns	*	ns	ns	ns
CO_2 × Irrigation			ns	ns	ns	ns	ns	ns	ns
T^e × Irrigation			ns	ns	ns	ns	*	ns	ns
$CO_2 \times T^e$ × Irrigation			ns	ns	ns	ns	*	*	ns

Three-way ANOVA was performed for lineal model on raw data. Significance: *$P \leq 0.05$, **$P \leq 0.01$ and ns indicates not significant. Comparison means by Duncan's test ($P \leq 0.05$) were shown for the significant interaction among treatments. Different letters indicate significant differences among data within the same factor or interaction. Amb= Ambient, Elev= Elevated; T^e= Temperature.
