# Supplementary material for: Interactional Effects of Climate Change Factors on the Water Status, Photosynthetic Rate, and Metabolic Regulation in Peach
Source: Front Plant Sci. 2020 Feb 28;11:43. doi: 10.3389/fpls.2020.00043 (PMC7059187; doi:10.3389/fpls.2020.00043)
Supplement: Supplementary file 10 [file Table_10.pdf]

Table S10. Summary of the genes involved in the transcriptomic changes in each rootstock-tissue at the different stress conditions.

Rootstocks	Stress	Roots	TOTAL root	Leaves	TOTAL leaf	(TOTAL) DIF
**GF677**	**Elev CO_2**	*SIP1 (1)*	1	*HAT22 (1)*	1	2
	**Elev Temperature**	*SDH, S6PDH, P5CS, DREB2, AREB2, HAT22 (6)*	6			6
	**Irrigation**	*SDH, S6PDH, P5CS, P5CR (4)*	1	*S6PDH, SIP1 , HAT22 (3)*	2	(7) 6
	**Interaction**	*SDH, S6PDH, SIP1, AREB2 (4)*		*SIP1, P5CS, P5CR, HAT22 (4)*	2	(8) 2
	TOTAL	***SDH, S6PDH , SIP1, P5CS , P5CR , DREB2, AREB2, HAT22***	**8**	***S6PDH, SIP1, P5CS, P5CR, HAT22***	**5**	**(13) 8**
**Adesoto**	**Elev CO_2**	*S6PDH, AREB2 (2)*		*SDH , P5CR, HAT22 (3)*	1	5
	**Elev Temperature**			*SDH (1)*		1
	**Irrigation**	*SDH (1)*		*P5CR (1)*		2
	**Interaction**	*S6PDH, PIP2 (2)*	1	*SDH, P5CS, P5CR, OAT (4)*	1	6
	**TOTAL**	***SDH, S6PDH, PIP2 , AREB2***	**4**	***SDH , P5CS, P5CR, HAT22, OAT***	**5**	**(9) 8**
**TOTAL TISSUES DIFFERENT**		***SDH, S6PDH , SIP1, P5CS, P5CR, DREB2, AREB2, HAT22, PIP2***	**9**	***SDH , S6PDH, SIP1, P5CS, P5CR , HAT22, OAT***	**7**	**(16) 10**
