# Supplementary material for: Interactional Effects of Climate Change Factors on the Water Status, Photosynthetic Rate, and Metabolic Regulation in Peach
Source: Front Plant Sci. 2020 Feb 28;11:43. doi: 10.3389/fpls.2020.00043 (PMC7059187; doi:10.3389/fpls.2020.00043)

**Figure S1**. Schematic representation of changes in the leaf-to-root ratio expressed on a logarithmic basis for different sugars and proline under stress conditions compared to control conditions in *Prunus* rootstocks (GF677 and Adesoto) budded with var. Catherina, after 23 days of treatment.

Log2 [((Leaf / Root) Stress) / ((Leaf / Root) Control)]

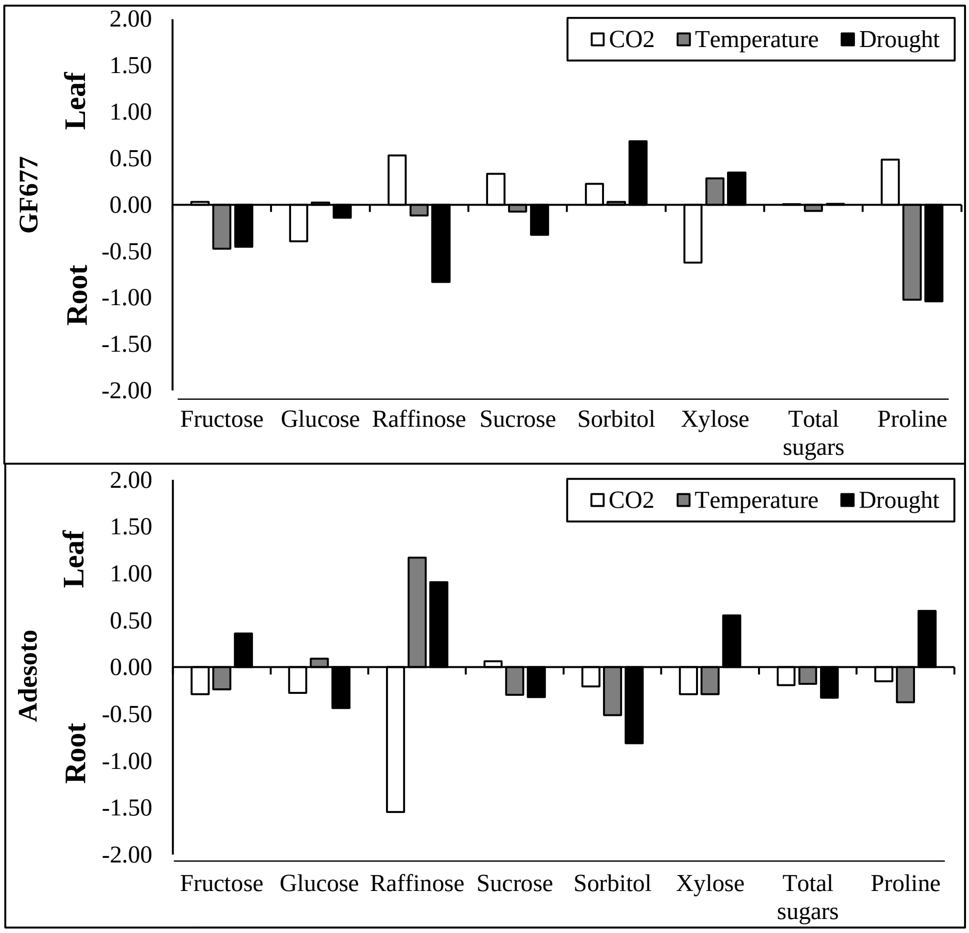

Supplement: Supplementary file 11 [file Image_1.pdf]
