# Supplementary material for: Interactional Effects of Climate Change Factors on the Water Status, Photosynthetic Rate, and Metabolic Regulation in Peach
Source: Front Plant Sci. 2020 Feb 28;11:43. doi: 10.3389/fpls.2020.00043 (PMC7059187; doi:10.3389/fpls.2020.00043)

Figure S2. Schematic representation of changes in root and leaf tissues expressed on a logarithmic basis for different sugars and proline under stress compared to control conditions in *Prunus* rootstocks (GF677 and Adesoto) budded with var. Catherina, after 23 days of treatment. [FC = log(2) (Stress/control)].

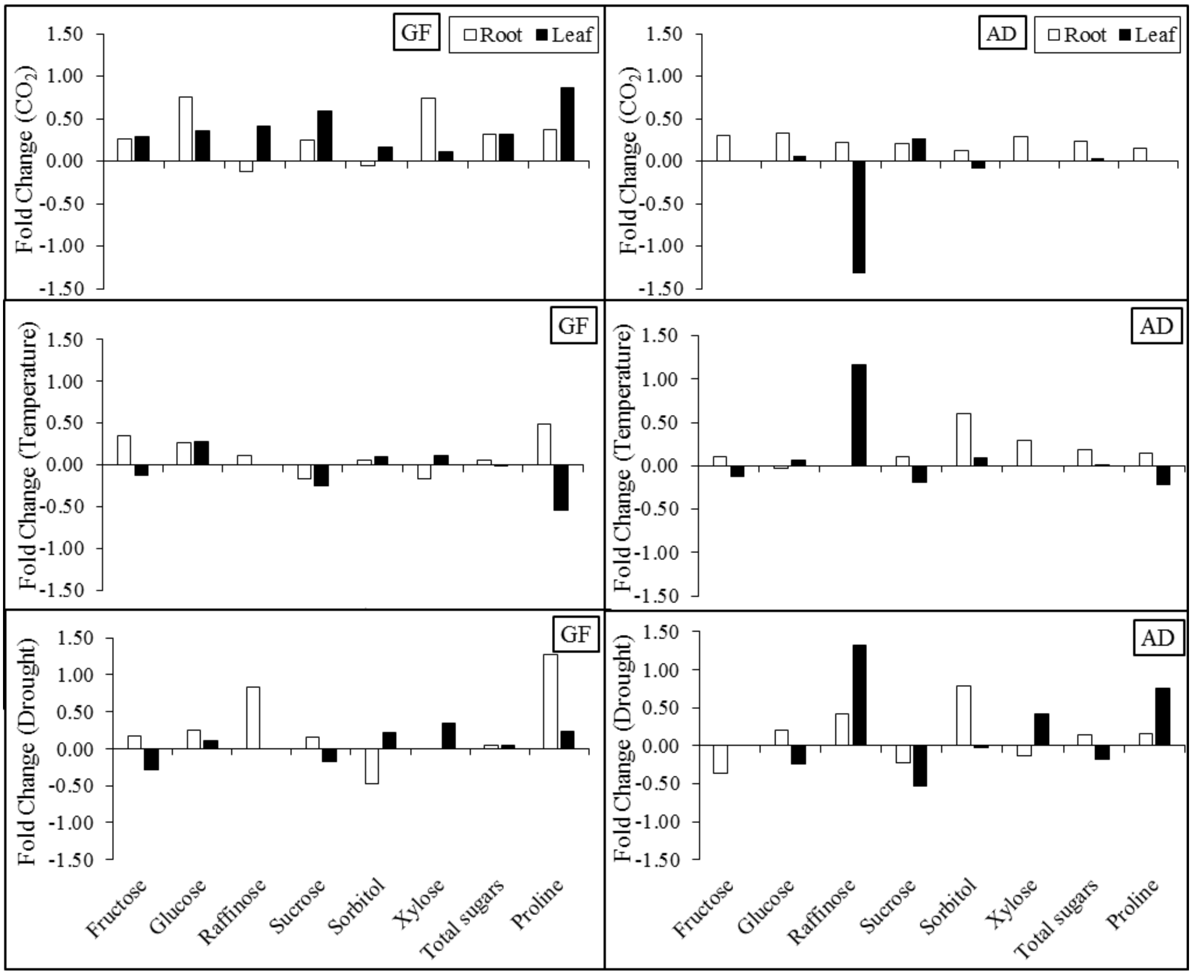

Supplement: Supplementary file 12 [file Image_2.pdf]
