# Supplementary material for: Interactional Effects of Climate Change Factors on the Water Status, Photosynthetic Rate, and Metabolic Regulation in Peach
Source: Front Plant Sci. 2020 Feb 28;11:43. doi: 10.3389/fpls.2020.00043 (PMC7059187; doi:10.3389/fpls.2020.00043)
Supplement: Supplementary file 13 [file DataSheet_1.pdf]

Supplementary Material

Interactional Effects of Climate Change Factors on the Water Status, Photosynthetic Rate, and Metabolic Regulation in Peach

Sergio Jiménez[1, 2†], Masoud Fattahi[1, 3†], Khaoula Bedis[1] , Shirin Nasrolahpour-moghadam[1,3], Juan José Irigoyen[4], Yolanda Gogorcena[1]*

[1]Laboratory of Genomics, Genetics and Breeding of Fruit Trees and Grapevine, Department of Pomology, Estación Experimental de Aula Dei-Consejo Superior de Investigaciones Científicas, 50059 Zaragoza, Spain.

[2]Present address: Bayer AG, CropScience Division, Development, Environmental Science, Building 6230, 40789 Monheim, Germany.

[3]Present address: Department of Agriculture, Shahrekord University, Shahrekord Iran.

[4]Universidad de Navarra, Dpto. de Biología Ambiental, Grupo de Fisiología del Estrés en Plantas, Unidad Asociada al CSIC (EEAD, Zaragoza e ICVV, Logroño), Facultad de Ciencias, Pamplona, Spain.

[†]Contributed equally

***Correspondence:**
Yolanda Gogorcena (aoiz@eead.csic.es)

1 Supplementary Figures and Tables

Uploaded separately as pdf files

1.1 Supplementary Figures

2

1.2 Supplementary Tables

10
